# GTX-11 attenuates lung fibrosis, inflammation and vascular remodeling in preclinical models of lung fibrotic disease

**DOI:** 10.3389/fphar.2025.1671132

**Published:** 2026-01-27

**Authors:** Ana Montes-Worboys, Javier Milara, Consol Farrera, Cristina Fernández-Asensio, Silvia Sánchez-Díez, Jaume Mercadé, Paula Montero, Inés Roger, María Molina-Molina, Eugènia Ruiz-Cánovas, Julio Cortijo

**Affiliations:** 1 Pulmonary Department, Unit of Interstitial Lung Diseases, University Hospital of Bellvitge, IDIBELL, University of Barcelona, Barcelona, Spain; 2 Biomedical Research Networking Centre on Respiratory Diseases (CIBERES) Health Institute Carlos III, Madrid, Spain; 3 Department of Pharmacology of the University of Valencia, Valencia, Spain; 4 GAT Therapeutics S.L., Barcelona, Spain; 5 Faculty of Health Sciences, Universidad Europea de Valencia, Valencia, Spain

**Keywords:** GTX-11, lung fibrosis, interstitial lung disease (ILD), TGFβ signaling, antifibrotic therapy, bleomycin, fibroblast activation, inflammatory cytokine

## Abstract

**Background:**

Fibrotic interstitial lung diseases (ILDs) are characterized by different degrees of inflammation and fibrosis of the lung parenchyma that are associated with progressive loss of breath, high morbidity and mortality. Current therapeutic options are limited, so there remains a significant need for effective and well-tolerated treatments. GTX-11 is an orally available small molecule in development for the treatment of fibrotic diseases. In this study, we aimed to assess the therapeutic potential of GTX-11 in different preclinical models of lung fibrotic disease.

**Methods:**

We assessed the activity of GTX-11 and its active metabolite, GTX-11m, in the bleomycin-induced pulmonary fibrosis model and *in vitro* in primary fibroblast cell cultures, including human normal lung fibroblasts (hNLFs) and ILD patient-derived fibroblasts.

**Results:**

In the murine model, GTX-11 treatment improved animal survival and significantly reduced lung fibrosis as measured by Ashcroft score and collagen deposition. GTX-11 also reduced the inflammatory cell count in bronchoalveolar lavage fluid and pro-inflammatory factors in lung tissue. Additionally, GTX-11 significantly improved lung vascular dysfunction and reduced pulmonary vascular remodeling. The preclinical anti-fibrotic effects of GTX-11 were comparable to, or in some cases exceeded, those of currently approved anti-fibrotic drugs used in clinical practice. *In vitro*, GTX-11m demonstrated anti-fibrotic and anti-inflammatory activity in hNLFs and ILD patient-derived fibroblasts. GTX-11m inhibited TGFβ-induced expression of key fibrotic markers and reduced fibroblast-to-myofibroblast transition and inflammatory cytokine production. The effects were consistent across the different tested ILD cultures and resulted from the prevention of SMAD2 and SMAD3 activation by TGFβ. The GTX‐11m anti-fibrotic and anti-inflammatory effects were comparable or better than nintedanib.

**Conclusion:**

Altogether, our studies reveal that GTX-11 is an effective antifibrotic both *in vivo* and *in vitro*, suggesting that GTX-11 has potential as a therapeutic option for fibrotic ILDs.

## 1 Introduction

Interstitial lung diseases (ILDs) comprise a heterogeneous group of lung diseases characterized by different degrees of inflammation and fibrosis of the lung interstitium and associated with substantial morbidity and mortality. Most fibrosing ILDs share common pathogenic pathways and similar clinical presentation, with progressive fibrosis, worsening respiratory symptoms, and decline in pulmonary function, accompanied by loss of quality of life (Amati et al., 2023). ILDs can be related to primary diseases, such as rheumatoid arthritis, sarcoidosis, scleroderma, or pneumonia; or to genetic or environmental factors. However, in several cases there is no identifiable cause, and it is classified as idiopathic pulmonary fibrosis (IPF). IPF is the most common form of fibrosing lung diseases, with a rising worldwide prevalence estimated to range from 7 to 1,650 per 100,000 persons (Shah Gupta et al., 2023) and a median survival time from diagnosis of 2–4 years (King et al., 2011; Richeldi et al., 2017). At the molecular level, pulmonary fibrosis is the result of repetitive alveolar damage and an abnormal repair process, which leads to scarring replacing healthy tissue and disrupting gas exchange. Transforming growth factor β (TGFβ) is considered the primary factor that drives fibrosis, as the sustained activation of this signaling pathway results in fibroblast to myofibroblast transition (FMT), excessive production of extracellular matrix (ECM) and inhibition of ECM degradation (Meng et al., 2016; Kim et al., 2018).

Despite advances in understanding fibrotic mechanisms, current ILD treatment options are very limited. Up until recently, pirfenidone and nintedanib were the only approved antifibrotic drugs for IPF treatment (Raghu et al., 2015), with nintedanib later extending its indication to progressive pulmonary fibrosis (PPF) as well (Raghu et al., 2022; Raman et al., 2023). Recently, nerandomilast received approval from the U.S. Food and Drug Administration for the treatment of IPF (U.S. Food and Drug Administration, 2025). However, this drug has not yet been evaluated in real-world populations outside of clinical trials, and its long-term efficacy and safety remain uncertain. Moreover, it shares overlapping adverse effects with existing antifibrotic drugs, such as gastrointestinal intolerance. Consequently, its overall clinical benefit in routine practice has yet to be established. On the other hand, pirfenidone and nintedanib have only been shown to slow disease progression and their associated adverse events lead to treatment discontinuation in approximately 20% of patients (Richeldi et al., 2014; Petnak et al., 2021). These frequent adverse effects include gastrointestinal disturbances such as nausea, diarrhoea, and loss of appetite; dermatological reactions such as rash and photosensitivity; and elevations in liver enzymes. The only existing curative treatment for IPF is lung transplantation, which is an alternative for only a small subset of patients due to its complexity and the restricted supply of donors (King et al., 2011; Richeldi et al., 2017). Therefore, there remains a significant need for effective and well-tolerated treatments for progressive fibrosing ILDs.

Some natural compounds capable of modulating the TGFβ pathway have gained interest as potential therapeutic candidates. Fucoxanthin (FX) is a carotenoid of marine origin with strong anti-inflammatory, anti-obesity and anticancer effects (Mohibbullah et al., 2022). FX has been reported to exert pulmonary, cardiac, renal and hepatic protection by inhibiting the TGFβ1-mediated fibrotic process. Particularly, in the lung, FX ameliorated bleomycin-induced fibrosis (Ma et al., 2017) and reduced the inflammatory phenotype (Li et al., 2020; Wu et al., 2021; Lee et al., 2022). Toxicity studies in animals and humans have confirmed FX’s safety, and it is FDA-approved as a nutritional supplement (Sayuti et al., 2023; Winarto et al., 2023). Moreover, it has been tested in human clinical trials suggesting pharmacological effects in metabolic syndrome (López-Ramos et al., 2023) and liver health (Sayuti et al., 2023). Yet, its efficacy is limited due to low stability and poor bioavailability (Koch et al., 2024). Once ingested, FX is converted to fucoxanthinol (FXol) in the gastrointestinal tract and to amarouciaxanthin A (ACX) in the liver. Therefore, the reported pharmacological properties of FX are probably exerted by its metabolites (Asai et al., 2004; Mok et al., 2018; Stiefvatter et al., 2021; Takatani et al., 2022), which also have direct antifibrotic activities (Li et al., 2021).

To overcome these limitations, GTX-11 has been developed. It is a pro-drug intended to improve the absorption and bioavailability of the active metabolite ACX (henceforth GTX-11m) over the values obtained with the oral administration of FX. We hypothesize that the administration of GTX-11 could be a suitable therapeutic alternative to the administration of the parental FX with a less complex pharmacokinetic profile. Comprehensive IND-enabling toxicological evaluations of GTX-11 (data not shown), including acute and subchronic studies in rodents and non-rodents conducted under GLP conditions, have demonstrated an excellent safety profile with no treatment-related adverse effects. In repeated-dose oral toxicity studies, no systemic or organ-specific toxicity was observed, and all hematological, biochemical, and histopathological parameters remained within normal ranges. Organ weights and histopathology, including cardiac, hepatic and dermal tissues, showed no test-item-related alterations. Furthermore, GTX-11 was non-mutagenic and non-phototoxic, indicating no genotoxic or cutaneous risks. These results establish GTX-11 as a well-tolerated compound, which has enabled it to reach clinical development for the treatment of fibrotic diseases. Additionally, we have recently proved the beneficial effects of GTX-11 on portal hypertension and liver fibrosis in a murine model of metabolic dysfunction-associated steatohepatitis (MASH) and in human liver tissues (Andrés-Rozas et al., 2025).

The objective of the present study was to evaluate the antifibrotic potential of GTX-11 in pulmonary fibrosis models. To this end, we characterized the effects of GTX-11 on lung hemodynamics, fibrosis, inflammation and vascular phenotype in a rat model of bleomycin-induced fibrosis. Additionally, we assessed the effects of GTX-11m in human normal lung fibroblasts (hNLFs) and in primary human fibroblasts derived from fibrotic ILD patients. Readouts for these *in vitro* studies included SMAD activation and fibroblast to myofibroblast transition markers, as well as fibrosis and inflammation markers. Our studies reveal that GTX-11 is an effective antifibrotic compound showing efficacy in an *in vivo* fibrosis model as well as in human culture systems.

## 2 Materials and methods

### 2.1 Reagents

GTX-11, used in animal studies, and GTX-11m, the active metabolite of GTX-11 used in the *in vitro* studies, were synthesized and purified by GAT Therapeutics. Nintedanib and pirfenidone were both purchased from MedChemExpress (HY-50904 and HY-B0673). Recombinant TGFβ1 was purchased from R&D systems (240-B-010).

### 2.2 Ethics statement

Experimentation and animal handling were performed in accordance with the guidelines of the Committee of Animal Ethics and Wellbeing of the University of Valencia (2020/VSC/PEA/0085 Valencia, Spain) following the ARRIVE guidelines (Kilkenny et al., 2010), and in accordance with EU Directive 2010/63/EU for animal experiments. All investigators understand the ethical principles. To minimize animal suffering, during invasive procedure/surgery and following procedures, adequate anesthesia and analgesia were administered. Animals that lost more than 40% of their body weight were sacrificed in accordance with internal protocols.

Adult human lung fibroblasts were obtained from lung biopsies of patients who underwent surgical biopsy for the diagnosis of a fibrotic lung disease (Raghu et al., 2011). The tissues were collected after obtaining written informed consent. All studies were approved by the Ethics Committee of Bellvitge Hospital (CEIC, ref. PR202/08) and were performed according to the Declaration of Helsinki guidelines of human sample and data collection, usage and storage.

### 2.3 Cell cultures

#### 2.3.1 Primary ILD fibroblasts obtention and culture

Lung biopsies were obtained from patients with forced vital capacity (FVC) > 50% and diffusing capacity of the lung for carbon monoxide (DLCO) > 35%, who underwent surgical biopsy at time of diagnosis of a fibrotic lung disease (histologically confirmed usual interstitial pneumonia), before initiating anti-fibrotic or other specific treatments. The study included three patient groups: IPF with a usual interstitial pneumonia pattern (n = 7, mean age 67 years, 100% male), fibrotic hypersensitivity pneumonitis (HP) (n = 5, mean age 64 years, 60% male), and sarcoidosis stage III–IV (n = 4, mean age 50 years, 75% male). Control lung biopsies were obtained from patients with pneumothorax or benign pleural pathology that required surgical treatment (n = 4, mean age 58, 100% male). Histological evaluation confirmed no interstitial abnormalities were present in these samples.

Primary human lung fibroblasts from ILD patients were isolated from lung biopsies, following previously optimized protocols (Merino et al., 2021). Briefly, lung tissue samples were maintained in a solution containing DMEM high Glucose with L-Glutamine (31966–021, Gibco Life Technologies) medium, HEPES (15630–056, Gibco Life Technologies), insulin, human transferrin and sodium selenite (ITS) (I-3146, Sigma-Aldrich) until processing. Then, samples were cut into small pieces and placed into six-well plates (Nunc Thermo Scientific, Waltham, MA, United States) with growth medium; DMEM supplemented with 10% inactivated FBS (10270, Gibco Life Technologies), penicillin (100 U/mL)/streptomycin (100 μg/mL) solution (15140–122, Gibco Life Technologies) and 25 μg/mL amphotericin B (A2942, Sigma-Aldrich). Cells were cultured at 37 °C in a humidified atmosphere of 5% CO_2_. Spindle-like primary fibroblasts started to grow out from tissue samples between days 2 and 3. Once the outgrowth of fibroblasts had occurred after 1–2 weeks, tissue samples were removed by aspiration, and cells were allowed to reach confluence. Upon reaching confluence, the cells presented fibroblast morphology, and were positive for vimentin (VIM) and negative for α-smooth muscle actin (αSMA) markers, indicating their mesenchymal origin. Fibroblasts at confluence were expanded by trypsinization, cryopreserved using standard methods, and passaged every 4–5 days at a 1:4 ratio. A PCR-mycoplasma test was routinely performed on all primary cultures. Cells between passages 4 and 7 were used in this study.

To evaluate GTX-11m effects on the TGFβ pathway, human lung primary fibroblasts were cultured in appropriate medium with 10% inactivated FBS, which was reduced to 2% once cells reached 80% confluence. Cells were stimulated with TGFβ1 at 5 ng/mL and GTX-11m 1 µM or DMSO 0.01% (D2653, Sigma) for 72 h. After the incubation period, cell supernatants were harvested for ELISA or multiplex assay and cell extracts were obtained for Western blot or qPCR analysis. Each condition was assayed in triplicates.

#### 2.3.2 Human normal lung fibroblasts culture

Human normal lung fibroblasts (hNLFs) (3310, ScienCell Research Laboratories), positive for fibronectin, were negative for HIV-1, HBV, HCV, *mycoplasma*, bacteria, yeast and fungi, and were grown following the manufacturer’s instructions. Briefly, cells were cultured in a humidified incubator at 37 °C with 5% CO_2_, in fibroblast basal media supplemented with FBS, fibroblast growth supplement, penicillin and actinomycin (2301, ScienCell Research Laboratories), and used before passage 15.

To evaluate the SMAD signaling pathway, hNLFs were cultured in the presence of GTX-11m at the indicated concentrations or 0.05% DMSO vehicle for 48 h. hNFLs were then stimulated with 0.5 ng/mL TGFβ1 for 30 min and extracted for Western blot analysis. To evaluate the subsequent effects on the TGFβ pathway, hNLFs were cultured in the presence or absence of TGFβ1 and GTX-11m at 1 μM, 2.5 µM and 5 µM or vehicle (DMSO 0.05%) for 48 h. To evaluate GTX-11m effects in comparison with nintedanib and pirfenidone, they were tested at concentrations referred by the manufacturer (1 µM and 1 mM, respectively). These concentrations are consistent with those used in previously published studies on human lung fibroblasts (Wollin et al., 2014; Molina-Molina et al., 2018). All tested compounds showed no cytotoxicity at the concentrations used. Cell supernatants were harvested for ELISA and cells were extracted for Western blot or qPCR analysis.

#### 2.3.3 Viability and proliferation assays

hNLFs proliferation was assessed by bromodeoxyuridine (BrdU) incorporation with a commercially available BrdU assay kit according to manufacturer’s instructions (QIA58, Sigma-Aldrich). Similarly, cell viability was checked by MTT using a commercially available kit according to manufacturer’s instructions (E-CK-A341, Elabscience).

#### 2.3.4 Wound healing assay

hNLF were seeded at a concentration of 30,000 cells/well in poly-L-lysine (A-005-C, Sigma-Aldrich) coated 24 well plates and rested overnight. Cells were then incubated with GTX-11m at 5 μM, or DMSO 0.05%, and with TGFβ1 at 0.5 ng/mL for 24 h. A 20 μL tip was used to make a straight scratch in each well. Detached cells were removed with two consecutive washes with Phosphate Buffered Saline (PBS) (L0615-500, Biowest). Fresh medium containing the same initial stimulations was added to each well. hNLFs were then returned to the incubator and left to migrate for 8 h.

Images of two to three different areas along each scratch were taken right after the damage (time zero) and after 8 h of migration using a Motic AE2000 optical microscope and a Motic 5.0 camera. The cell-free areas were measured using ImageJ software. Wound closure is expressed as a difference of cell-free areas at time 0 and after 8 h of migration, in percentages. Each condition was assayed in quintuplicate.

### 2.4 Animal studies

#### 2.4.1 Bleomycin rat fibrosis model and sample collection

Animals were housed with free access to water and food under standard conditions: relative humidity 55% ± 10%; temperature 22 °C ± 3 °C; 15 air cycles/per hour; 12/12 h Light/Dark cycle. Biosis cages (2154F, Tecniplast), beds from Sodispan biotech (ECO7/GRADE7), and Tecklad Global Rodent diet (Inotive) were used. All animals were given 15 days to acclimatize to the housing condition before the start of the experiment. The animal sex, species, strain and age were selected according with the Use of Animal Models for the Preclinical Assessment of Potential Therapies for Pulmonary Fibrosis guidelines (Jenkins et al., 2017) and relevant revisions of pulmonary hypertension animal models (Ryan et al., 2011; Dignam et al., 2022).

Pathogen-free male Wistar rats (Charles Rivers), 12 w. o., weight between 220 and 230 g, were randomly allocated to 5 treatment homogeneous groups (n = 12 each group), based in previous findings (Almudéver et al., 2013), on the day before treatment. The primary outcome was established as the 50% reduction of the Ashcroft score fibrosis index by GTX-11. The sample size was calculated for an effect size f of 1.8, α error of 0.05 and power of 0.96, using one tailed analysis of variance (ANOVA). Sample size for hemodynamic imaging and tissue-based assays were determined based on sample availability and technical needs as indicated in figure legends.

On day 1, rats were anaesthetized using 5% induction isoflurane and a single intratracheal dose of bleomycin (3.75 U/kg dissolved in 20 μL of saline) (Mylan Pharmaceuticals, S.L) or sham (saline) (190/12606059/1013, B. Braun) was administered. Therapeutic treatments, orally administered from day 10 to day 28 (once a day), were as follows: (i) sham + vehicle; (ii) bleomycin + vehicle; (iii) bleomycin + GTX-11 (8 mg/kg/day); (iv) bleomycin + GTX-11 (2 mg/kg/day); (v) bleomycin + nintedanib (50 mg/kg/day). GTX-11 was administered in sunflower oil at 1.25 mL/kg and nintedanib was administered in 40% PEG300 (202371, Merck) at 0.85 mL/kg. Nintedanib was administered at 50 mg/kg, a dose commonly used dose in rodent studies (Yu et al., 2022; Ding et al., 2024; Tanriverdiyeva et al., 2024) which slightly surpasses the human therapeutic exposure of 150 mg twice daily when applying standard allometric scaling. GTX-11 doses (2 and 8 mg/kg) were selected based on previous studies in liver fibrosis models (Andrés-Rozas et al., 2025).

On day 27, magnetic resonance imaging of the lungs (micro-PET-CT) was measured in 5 animals per group. On day 28, all animals were anesthetized, and the right ventricular systolic pressure (RVSP) was measured by right heart catheterization. Lung inflammation and endothelial function were also assessed in an *In Vivo* Imaging System (IVIS) on day 28 in 4 anesthetized animals of each group. At the end of hemodynamic measures, animals were euthanized by a 100 mg/kg intraperitoneal injection of sodium pentobarbital followed by exsanguination and bronchoalveolar lavage fluid (BALF) was obtained to determine inflammatory cell populations in the alveolar lumen. After that, lungs and heart were removed *en bloc,* photographed and stored to perform histology, protein and gene expression studies. Specifically, the tissue for histology was washed with saline solution and fixed in formalin for subsequent dehydration. For protein analysis, the tissue was washed, cut, and stored dry at −80 °C. For RNA analysis, the tissue was washed, minced, and preserved in RNAlater (AM7020, Thermo Fisher) to maintain the integrity of the genetic material, and then stored at −80 °C.

#### 2.4.2 Magnetic resonance imaging (MRI) of the lungs

Lung imaging by magnetic resonance was performed as previously described (Mahmutovic Persson et al., 2020). The acquisition of images was conducted using the MRS*DRYMAG 3017-3.0T MRI system (MR Solutions), with animals placed under anesthesia using a Cyprante Keighleit anesthesia machine and a flow-meter rotameter regulating oxygen supply. The inhaled anesthesia consisted of two phases: induction phase, which is carried out at 3%–4% isoflurane (Zoetis-IsoFLo® 100% w/w, Baxter SL) and 100% oxygen (Oximesa-Nippon Gases, Spain); and maintenance phase, in which the concentration of isoflurane is lowered to 1%–2.5%, maintaining oxygen at 100%. Image acquisition was carried out using the Preclinical SCAN software (MR Solutions), applying predefined MRI protocols adjusted to suit the specific experimental needs. A whole-body FLASH-3D sequence of 20 min duration was employed, consisting of 128 slices, a field of view (FOV) of 60 × 80 mm, slice thickness of 0.39 mm, and a matrix of 256R x 128P x 1S. Images were reconstructed in real time and were further enhanced in quality with “Retro” sequence. It generated images with a slice thickness of 1 mm, FOV of 80 × 80 mm, and a matrix of 256R x 256P x 1S across 11 slices. From these images, lung volume (mm^3^) was calculated in the slice with the largest volume using the MRS*Preclinical Scan-Multimodality Imaging Software. Heart hypertrophy was calculated as the increase of total wall thickness (WT). Total WT was quantified from selected MRI heart slices by measuring the ratio between the WT of the right heart ventricle (RHV) and the left heart ventricle (LHV). WT percentage was calculated as the ratio of (external diameter (ED) – internal diameter (ID))/ED x 100, as previously described (Ruffenach et al., 2016; Rol et al., 2017).

#### 2.4.3 IVIS imaging

Acute pulmonary inflammation was assessed in an IVIS system using whole animals. Approximately 24 h prior to imaging, the vascular imaging dye IVISense Vascular NP 680 Fluorescent Nanoparticles (AngioSPARK) (NEV10149, Perkin Elmer) was injected via the tail vein. During image acquisition, animals were anesthetized using an anesthesia inhalation system (Harvard Apparatus, Holliston, MA) with a mixture of 2% isoflurane and 2 L/min oxygen and images were captured using a whole body IVIS Lumina X5 imaging system (Perkin Elmer). Fluorescence was quantitated in the lung region and expressed as Radiant Efficiency, i.e., the quotient of emission light radiance (photons/sec/cm2/str) and excitation light power (µW/cm2).

#### 2.4.4 BALF cellular content

BALF was obtained after exsanguination of the lungs, using 3 mL of media and 3 lavages of 1 mL. Total number of cells was quantified in a hemocytometer, and differential cell counting in 100 µL of BALF was done after cell spin centrifuge and Giemsa staining as previously described (Cortijo et al., 2001).

#### 2.4.5 Right heart remodeling and hypertrophy

For hemodynamic measures, animals were anesthetized using isoflurane at 5% in the induction phase and at 2% during maintenance. Buprenorphine (Zoetis) 0.1 mL/kg and meloxicam (Zoetis) 0.3 mL/kg were administered intraperitoneally as analgesics.

RVSP was measured by right heart catheterization as previously reported (Almudéver et al., 2013). Briefly, the right jugular vein was cannulated with a small silicone catheter (BPE-T50 Polyethylene tubing for 22ga swivels; Salomon Scientific) containing heparin saline solution (10 UI/mL of heparin in 0.9% saline), to reach the right ventricle (RV) under the guidance of the pressure tracing. After 20 min of stabilization, RVSP was recorded using a miniature pressure transducer (TSD104A, BIOPAC Systems, Inc.) digitized by a BIOPAC MP100 data acquisition system.

The RV wall of the heart was dissected *free* and weighed along with the left ventricle wall plus septum (LV + S), and the resulting weights are reported as RV/LV + S ratio to provide an index of right ventricular hypertrophy.

#### 2.4.6 Histological studies

Lung histology was conducted as previously reported (Milara et al., 2018a; Milara et al., 2018b). Here, tissue sections (4 μm thickness) were stained with Masson’s trichrome (256692, Panreac) to analyze both collagen deposition and pulmonary vascular remodeling. Lung tissue analysis, histology and pathological scoring were performed blinded to treatment.

Fibrosis severity was assessed using the Ashcroft scoring system, where a pathologist evaluated the severity of lung fibrosis on a scale from 0 (normal lung) to 8 (total fibrotic obliteration of tissue) (Ashcroft et al., 1988; Raghu et al., 2011).

To study pulmonary vascular remodeling, the degree of muscularization of intra-acinar pulmonary vessels was determined. Lung sections stained with Masson’s Trichrome were analyzed using a morphometric system (MetaMorph software, Molecular devices PA, United States). In each animal, 25–40 intra-acinar arteries with an external diameter between 20 and 50 μm were measured for internal area (IA), external perimeter (EP) and external area (EA). The absolute wall area (WA) was calculated with the following formula: WA = EA-IA. The pulmonary artery wall thickness was calculated by dividing the WA by the EP (James et al., 1988). All measurements were made by the same observer.

#### 2.4.7 Lung hydroxyproline determination

Rat lung tissue hydroxyproline concentration was determined using a commercially available Hydroxyproline Assay Kit (MAK008, Sigma Aldrich), according to the manufacturer’s protocol.

#### 2.4.8 Biochemistry and hematologic analysis

Hematologic and biochemical analysis were done using commercially available apparatus and kits according to manufacturer’s instructions (BC-30 Auto Hematology Analyzer, Mindray animal care), SMT-120V Chemistry Analyzer and Biochemistry reagent disc 24 Comprehensive Test Plus (Seamaty).

### 2.5 Sample analysis

#### 2.5.1 Western blot

Western blot analysis was used to detect changes in protein expression in rat lung tissue and in cell extracts from hNLFs and ILD fibroblasts. Lung tissue samples were homogenized using a TissueLyser device (Qiagen) and lysed on ice with lysis buffer (20 mM Tris base, 0.9% NaCl, 0.1% Triton X-100, 1 mM EDTA, 1 mM DTT and 1 mg/mL pepstatin A) supplemented with protease inhibitor cocktail (11697498001, Roche Diagnostics). hNLFs were lysed with RIPA buffer containing Halt inhibitors cocktail (78442, ThermoFisher). Proteins were quantified in each sample to ensure equal protein loading. 15 μg of protein were run onto an acrylamide gel at 100 V for 1 h and transferred from the gel to polyvinylidene difluoride (PVDF) or nitrocellulose membranes (PB7320, Invitrogen), for lung extracts or cell extracts respectively. PVDF membranes were blocked with 5% dry skimmed milk in PBS (BP665-1, ThermoFisher) containing 0.1% Tween 20 while nitrocellulose membranes were blocked in 5% BSA (BP9703, ThermoFisher) in TBS containing 0.1% Tween 20.

Membranes were then probed with antibodies described on Supplementary Table S1. The amount of detected protein was normalized to total rat β-actin or human glyceraldehyde-3-phosphate dehydrogenase (GAPDH), actin, α-tubulin (αTUB) or vinculin (VIN), as stated. HRP-conjugated secondary antibodies were used together with enhanced chemiluminescence reagents (34580, ThermoFisher) for final detection. Densitometry of target and reference signals were performed using the ImageJ 1.42q software or Image Studio Lite v5.2.5 and results of target protein expression were expressed as the ratio to endogenous controls.

#### 2.5.2 qPCR

Lung tissue was homogenized using the TissueLyser system (Qiagen) followed by total RNA isolation using TriPure® Isolation Reagent (1166716500, Roche). In the cDNA synthesis step, 300 ng of tissue RNA were used with the TaqMan reverse transcription reagents kit (N8080234, Applied Biosystems). cDNA amplification was done with predesigned specific primers (Supplementary Tables S2, S3) and Universal Master Mix (4369016, Applied biosystems) in a 7900HT Fast Real-Time PCR System (Applied Biosystems). Fold changes in expression were then calculated as the difference between the threshold cycle (Ct) value of target genes and β-actin (ΔCt), designated 2^−ΔCt^.

In hNLFs culture, total RNA was extracted using TRIzol (15596018, Invitrogen) as recommended by the manufacturer. Here, 1 μg of RNA was subjected to reverse transcription using M-MLV Reverse transcriptase [H-] Point Mutant (M3683, Promega), oligo (dT)15 primer and PCR nucleotide mix (U1420, Promega). Quantitative real-time PCR (qPCR) was performed using the Power SYBR Green Reagent Kit (4368706, Applied Biosystems) following the manufacturer’s recommendations. Data was expressed as mRNA levels relative to *GAPDH* expression or a composite of housekeeping genes.

In ILD patient’s derived fibroblasts, total RNA was isolated from cultured cells using the Qiagen RNeasy Mini Kit (74134, Qiagen) according to the manufacturer’s recommendations. Samples were digested with DNase I (79254, Qiagen) to remove contaminating genomic DNA and 1 μg of RNA was reverse-transcribed using the iScript cDNA synthesis kit (1708840, Bio-Rad) with oligo deoxythymidine and random hexamer primers. Quantitative real-time PCR (qPCR) was performed using SYBR Green PCR Master Mix and specific sequence primers. The relative gene expression of each targeted gene was normalized by the corresponding housekeeping genes (*ACTB*, *HPRT* and *RNA18S*) Ct value using the comparative Ct method (ΔΔCt methods).

A list of the primers used in this assay can be found on Supplementary Tables S2, S3.

#### 2.5.3 ELISA and multiplex protein assays

Levels of secreted proteins in supernatants from ILD patient’s derived fibroblasts were analyzed as follows. Tenascin C (TNC), matrix metalloproteinase 1 (MMP1), interleukin 6 (IL6), IL8 and tumor necrosis factor α (TNFα) were all analyzed with ProcartaPlex assays (Human ProcartaPlex Mix&Match, ThermoFisher) using the Luminex xMAP technology in a MAGPIX instrument, following the manufacturer’s instructions. To analyze the levels of monocyte chemoattractant protein 1 (MCP1), a DuoSet ELISA kit (DY279, R&D Systems) was used according to the manufacturer’s instructions.

### 2.6 Statistical analysis

All data is presented as mean ± standard deviation (SD) unless otherwise stated. Statistical analysis of results was performed by ANOVA or mixed-effect analysis as appropriate, followed by Dunnett’s multiple comparison test to detect differences among treatments. To compare data without normal distribution, Friedman test and Dunn’s multiple comparison test were applied. All statistical analyses were performed with GraphPad Prism 10 Software. A *p*-value of <0.05 was considered statistically significant.

To compare qPCR and Western blot data from hNLFs (Figure 4; Figure 6; Supplementary Figure S4; Supplementary Figure S6), the values were normalized as follows: the intensity of each experiment (i_e_) was calculated by determining the average expression value between the vehicle-treated control and the TGFβ-treated control. The intensities of individual experiments were then normalized by the overall mean intensity value of all the experiments (i_m_), obtaining as a result a normalization factor (i_m_/i_e_) for each experiment, which was later multiplied by the individual expression values of that experiment.

Conversely, cell viability and proliferation data from hNLFs was baseline normalized or rescaled to vehicle-treated control (Figure 4; Supplementary Figure S4). This normalization to baseline was also applied to qPCR and protein secretion data from patient-derived fibroblasts (Figure 5; Supplementary Figure S5).

## 3 Results

### 3.1 GTX-11 treatment improves survival and alleviates lung fibrosis in a rat model of bleomycin-induced fibrosis

**FIGURE 1:**
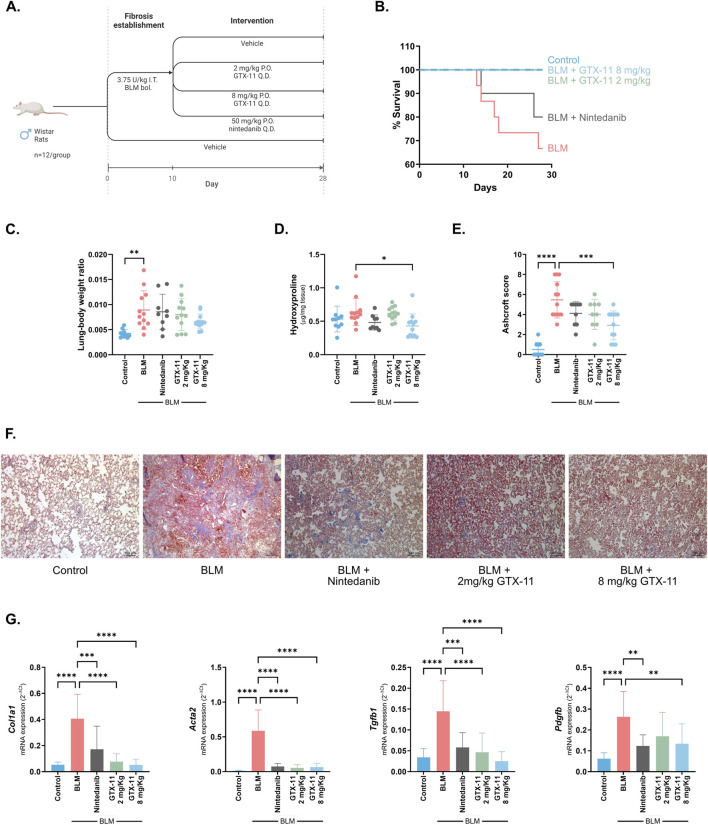
GTX-11 improves fibrotic symptoms and outcome in a rat model of bleomycin-induced lung fibrosis. **(A)** Therapeutic approach used in the bleomycin-induced fibrosis model. Bleomycin (BLM) was administered intratracheally (I.T.) to Wistar rats at day 1 and lung fibrosis was established by day 10. From day 10 to day 28, daily (Q.D.) oral administrations (P.O.) of the different treatments were as follows: vehicle to the control (n = 10) and bleomycin (n = 15) groups, and GTX-11 at 8 mg/kg/day (n = 12), GTX-11 at 2 mg/kg/day (n = 13) and Nintedanib at 50 mg/kg/day (n = 10) to the corresponding groups. **(B)** Kaplan-Meier survival plot of the different treatment groups. **(C)** Relative lung to body weight (mg/mg). **(D)** Hydroxyproline content was measured in homogenized lung tissue and is depicted as μg of hydroxyproline per mg of tissue. **(E)** Masson’s trichrome staining of lung sections was evaluated by a pathologist to assess the Ashcroft score in each animal. **(F)** Representative images (10X) of a lung section from each treatment group. Scale bars 100 μm. **(G)** Gene expression of relevant fibrotic markers *Col1a1*, *Acta2*, *Tgfb1* and *Pdgfb* in lung tissue. Individual data points or bars with mean values ±SD are shown. One way ANOVA with Dunnett’s multiple comparison correction was performed in all graphs. *p < 0.05, **p < 0.01, ***p < 0.001, ****p < 0.0001.

GTX-11 therapeutic potential was assessed in a well-established bleomycin-induced lung fibrosis model. In this model, lung fibrosis is fully developed 10 days after the initial bleomycin instillation, and therapeutic treatments are administered daily from day 10 to the end of the procedures on day 28 (Peng et al., 2013). GTX-11 was orally administered at two different doses (8 mg/kg and 2 mg/kg), while nintedanib was administered at a consensus dose (Figure 1A). The survival rate in the fibrotic group of bleomycin-treated rats was 67%, while the nintedanib group achieved 80% survival. Remarkably, survival rate was 100% in the two GTX-11 treated groups (Figure 1B).

Lung-to-body weight ratio was significantly increased in the bleomycin treated group compared to the control group (p = 0.001). GTX-11 at 8 mg/kg and 2 mg/kg appeared to reduce the elevated relative organ weight by 49% and by 19%, respectively (p = 0.158, p = 0.854) (Figure 1C). *In vivo* lung imaging by MRI confirmed the increase in lung volume produced by bleomycin (p = 0.033), and suggested again a reduction in the GTX-11 8 mg/kg group (p = 0.120), while GTX-11 at 2 mg/kg and nintedanib exerted milder or no apparent effects (p = 0.516, p = 0.999) (Supplementary Figures S1A,B). Although no statistically significant differences were found when data was analyzed collectively by one-way ANOVA, a separate *post hoc *t-test indicated a significant reduction at GTX-11 8 mg/kg (p = 0.028).

Hydroxyproline content and Ashcroft score in the lung confirmed the induction of fibrosis by bleomycin (p = 0.384, p < 0.001). GTX-11 at 8 mg/kg significantly reduced both parameters (p = 0.011, p < 0.001), while the 2 mg/kg dose or nintedanib showed similar although not significant reduction tendencies (p = 0.991, p = 0.071; nintedanib p = 0.111, p = 0.107) (Figures 1D–F). Further, macroscopic observation of the lungs showed patched fibrotic lesions in the bleomycin group. These lesions were reduced by GTX-11 at both concentrations and by nintedanib to a lesser extent (Supplementary Figure S1C). qPCR analysis of critical fibrotic markers in the lung–collagen 1 (*Col1a1*), α-smooth muscle actin (*Acta2*), *Tgfb1*, platelet-derived growth factor (*Pdgfb*) and collagen 3 (*Col3a1*) – revealed a robust induction by bleomycin (p < 0.001 for all markers) and consistent inhibition by both GTX-11 doses (2 mg/kg p < 0.001, except *Pdgfb* p = 0.062 and *Col3a1* p = 0.016; 8 mg/kg p < 0.001, except *Pdgfb* p = 0.005 and *Col3a1* p = 0.006), as well as by nintedanib (p < 0.001, except *Pdgfb* p = 0.005) (Figure 1G; Supplementary Figure S2A). While nintedanib did not show effect in *Tnc*, fibronectin (*Fn*), or in the metalloprotease system (*Mmp2*, *Mmp12*, Tissue Inhibitor of Metalloproteinase 1 (*Timp1*)) (p = 0.428, p = 0.704, p = 0.504, p = 0.793, p = 0.673), GTX-11 consistently reverted their induction (2 mg/kg p = 0.009, p = 0.002, p = 0.007, p = 0.020, p = 0.001; 8 mg/kg p = 0.008, p < 0.001, p = 0.004, p = 0.065, p < 0.001). Protein levels of collagen 1 (COL1), collagen 3 (COL3) and FN were also assessed by Western blot, showing similar tendencies as those of the qPCR (Supplementary Figure S2B).

### 3.2 GTX-11 treatment improves the inflammatory component in a rat model of bleomycin-induced fibrosis

**FIGURE 2:**
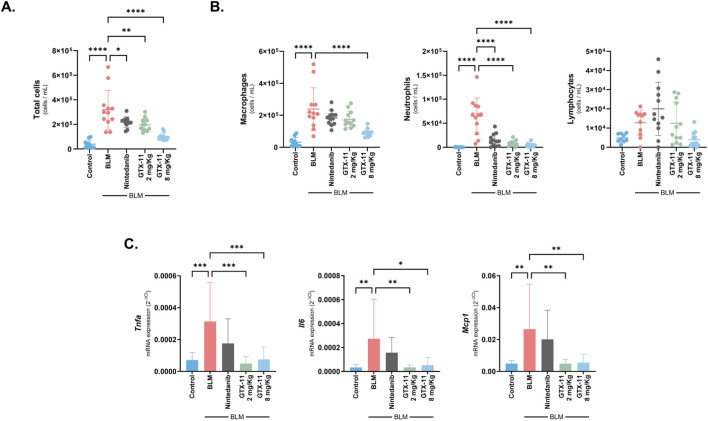
GTX-11 reduces BALF cellular infiltration and lung inflammatory gene expression in bleomycin-induced fibrosis. **(A)** BALF total cell infiltrate counts. **(B)** BALF differential cell counting of macrophages, neutrophils and lymphocytes. **(C)** Gene expression of relevant inflammatory markers *Tnfa*, *Il6* and *Mcp1* in lung tissue lysates. Individual data points or bars with mean values ±SD are shown. One way ANOVA with Dunnett’s multiple comparison correction was performed in all graphs. *p < 0.05, **p < 0.01, ***p < 0.001, ****p < 0.0001.

BALF total and differential cell counting served to assess alveolar inflammation, as well as to broadly characterize it. Bleomycin significantly increased total BALF cells (p < 0.001), composed primarily of macrophages and neutrophils, and a minority of lymphocytes. Compared to the bleomycin group, GTX-11 reduced the BALF total cell count by reducing these three populations at both doses, being 8 mg/kg more effective than 2 mg/kg (p < 0.001, p = 0.002). Nintedanib showed a clear reduction only in the neutrophil population (p < 0.001) (Figures 2A,B). qPCR analysis of key inflammatory markers commonly associated with lung fibrosis revealed a strong induction following bleomycin treatment. Conversely, GTX-11 treatment significantly inhibited the expression of *Tnfa*, *Il6* and *Mcp1* at both doses (2 mg/kg p < 0.001, p = 0.006, p = 0.004; 8 mg/kg p < 0.001, p = 0.013, p = 0.006), while nintedanib did not cause comparable reductions (p = 0.098, p = 0.404, p = 0.766) (Figure 2C).

### 3.3 GTX-11 treatment improves cardiovascular and pulmonary endothelial dysfunctions in a rat model of bleomycin-induced fibrosis

**FIGURE 3:**
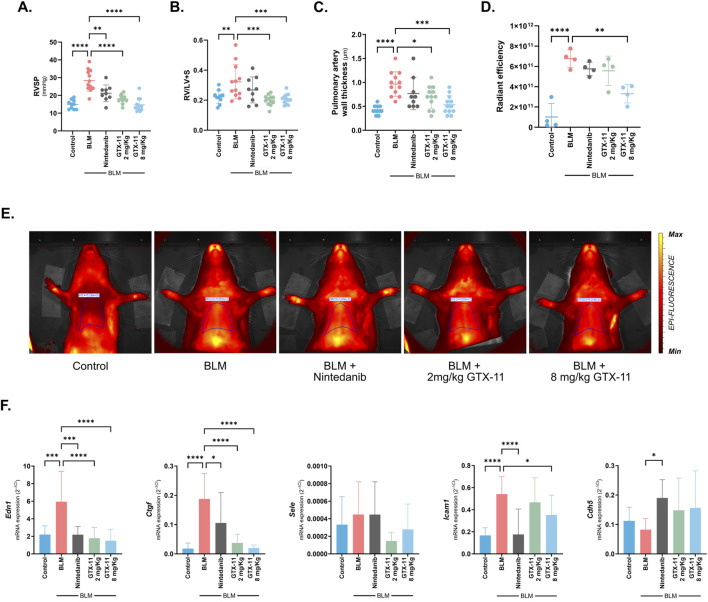
GTX-11 restores cardiovascular affectation and improves the pulmonary endothelial dysfunction induced by bleomycin. **(A)** RVSP, measured by right heart catheterization. **(B)** Fulton’s index indicating RV hypertrophy is reported as RV weight divided by the combined weight of the LV and the intraventricular septum. **(C)** Pulmonary artery wall thickness is reported as artery wall area normalized by perimeter. **(D)** Relative radiant efficiency measured in the IVIS system [(p/s/cm^2^/sr)/(μW/cm^2^)] (n = 4). High values indicate compromised lung vascular barrier and increased inflammation. **(E)** Representative images of animals’ relative fluorescence after the tracer injection obtained in the IVIS system. Yellow areas indicate maximal tissue permeability. **(F)** Gene expression of relevant endothelial markers *Edn1, Ctgf, Sele, Icam1* and *Cdh5* in lung tissue lysates. Individual data points or bars with mean values ±SD are shown. One way ANOVA with Dunnett’s multiple comparison correction was performed in all graphs. *p < 0.05, **p < 0.01, ***p < 0.001, ****p < 0.0001.

Cardiovascular affectations are prevalent and relevant comorbidities associated with lung fibrosis, greatly contributing to morbidity and mortality in this disease. In the same manner, bleomycin-induced lung fibrosis leads to severe affections to the cardiovascular system. Accordingly, we found a significant increase in RVSP in the bleomycin group (p < 0.001). GTX-11 at 8 mg/kg completely restored the RVSP to healthy control values (p < 0.001), while nintedanib and GTX-11 at 2 mg/kg significantly decreased it (p = 0.001, p < 0.001) (Figure 3A). Right ventricular hypertrophy was also confirmed in the bleomycin group (p = 0.006). GTX-11 completely prevented it, restoring the Fulton index (RV/LV + S) to that of the healthy control group (p < 0.001) (Figure 3B). MRI analysis of the heart revealed right ventricular hypertrophy in the bleomycin-treated group, indicated by an increased total ventricular wall thickness (p = 0.007). Treatment with GTX-11 at 8 mg/kg effectively reduced it (p = 0.001) (Supplementary Figure S1B). The bleomycin group evidenced an increase in pulmonary artery wall thickness (p < 0.001). This arterial muscularization was significantly reduced by GTX-11 at 8 mg/kg (p < 0.001) and 2 mg/kg (p = 0.021), while nintedanib showed a similar, albeit not significant, tendency (p = 0.179) (Figure 3C). Levels of compromised lung vascular barrier and lung inflammation were measured by *in vivo* imaging with AngioSPARK 680 fluorescent tracer. Here, the increase in vascular permeability observed in the bleomycin group (p < 0.001) was significantly reduced by 60% with GTX-11 at 8 mg/kg (p = 0.001), while GTX-11 at 2 mg/kg and nintedanib showed milder, non-significant reductions (p = 0.367, p = 514) (Figures 3D,E). These findings were further validated by qPCR analysis of endothelial markers in the lung. The vascular dysfunctional markers endothelin-1 (*Edn1*) and connective tissue growth factor (*Ctgf*), as well as the vasculature inflammation mediator intercellular adhesion molecule 1 (*Icam1*), were all found to be induced by bleomycin (p < 0.001) and significantly reduced by GTX-11 at 8 mg/kg (p < 0.001, except *Icam1* p = 0.042). Conversely, the basal expression of the cell adhesion molecules E-selectin (*Sele*) and VE-cadherin (*Cdh5*) were only partially and non-significantly restored by GTX-11 at the same dose (Figure 3F) (p = 0.460, p = 0.130). Finally, GTX-11 at both doses was also capable of normalizing the expression levels of both the ROS producing enzyme (*Nox4*) and the vascular endothelial growth factor (*Vegf*) (p < 0.001), while it was unable to restore CD31 (*Pecam1*) values back to control expression levels (Supplementary Figure S3) (2 mg/kg p = 0.863, 8 mg/kg p = 0.100).

### 3.4 GTX-11m reduces TGFβ activation in hNLFs

Fibroblast activation and differentiation into myofibroblasts are major hallmarks of human fibrotic diseases and they critically determine the course of the disease. Hence, hNLFs were used to validate the efficacy of GTX-11 *in vitro*. Although multiple factors are known to activate fibroblasts, the TGFβ pathway stands as the most important driver of the fibrotic cascade. TGFβ is produced and secreted in an inactive form and subsequently activated through both integrin-dependent and independent mechanisms, enabling it to bind to its receptor TGFBR2. This binding triggers sequential activation of TGFBR2, TGFBR1, and the downstream signaling mediators SMAD2 and SMAD3. Phosphorylated SMAD2/3 then form heterodimers with SMAD4 and translocate to the nucleus, where they promote the activation of an extensive transcription program of extracellular matrix proteins, growth factors and other profibrotic mediators. Tight regulation of this pathway is essential for tissue homeostasis, whereas its dysregulation represents a key driver of fibrosis (Deng et al., 2024). We have recently proved that GTX-11m is able to modulate SMAD activation by TGFβ1 in liver LX2 cells (Andrés-Rozas et al., 2025). Moreover, fucoxanthin, the natural carotenoid precursor of GTX-11m, is able to modulate this pathway in pulmonary fibroblasts (Ma et al., 2017).

**FIGURE 4:**
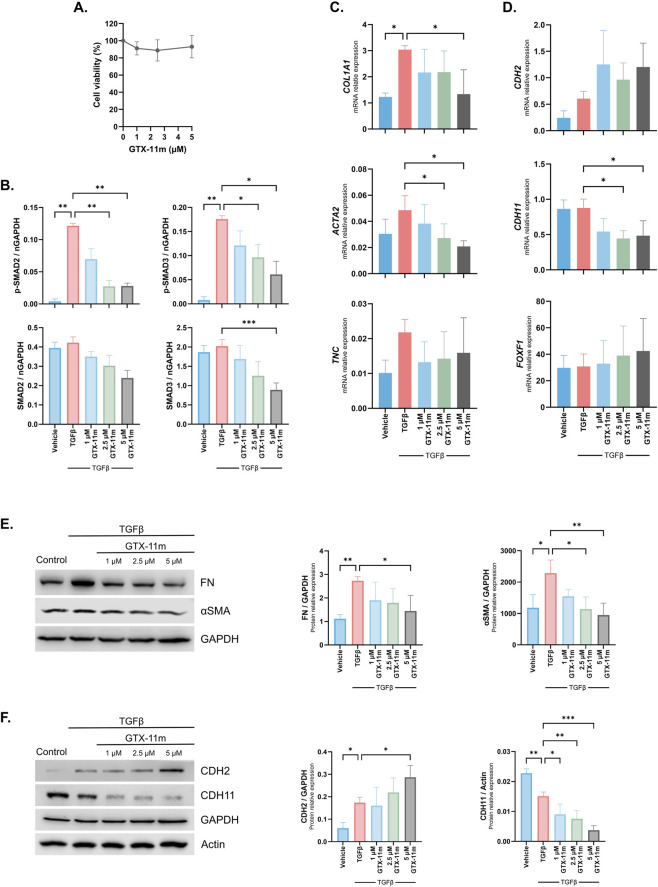
GTX-11 weakens TGFβ signaling, reduces fibrotic markers and prevents fibroblast-to-myofibroblast transition in hNLFs. **(A)** Cell viability was assessed in hNLF after 48 h of treatment with GTX-11m at the indicated concentrations by an MTT assay. Data is represented as percent change relative to vehicle (n = 4). **(B)** SMAD2 (Ser465/Ser467) and SMAD3 (Ser423/425) phosphorylation and total amounts were measured by Western blot after TGFβ1 and GTX-11m treatments (n = 3). **(C)** Gene expression levels of relevant fibrotic markers *COL1A1*, *ACTA2* and *TNC* after TGFβ1 and GTX-11m treatment for 48h were assessed by qPCR (n = 4). **(D)** Changes in the expression of relevant FMT markers *CDH2*, *CDH11* and *FOXF1* after GTX-11m treatment for 48h in hNLFs, as assessed by qPCR (n = 4). **(E)** Protein levels of FN and αSMA after GTX-11m treatments in hNLFs, as detected by Western blot. Left panel, representative images; middle and right panels, quantitation of three independent experiments. **(F)** Changes in protein levels of CDH2 and CDH11 after GTX-11m treatments in hNLFs, as detected by Western blot. Left panel, representative images; middle and right panels, quantitation of three independent experiments. Bars show mean ± SD. One way ANOVA with Dunnett’s correction for multiple comparison was performed in all graphs, except for cell viability assessment, where Friedman test with Dunn’s multiple comparison test was applied. *p < 0.05, **p < 0.01, ***p < 0.001.

In hNLFs, GTX-11m treatments at the indicated concentrations did not affect cell viability nor proliferation (Figure 4A; Supplementary Figure S4A). Importantly, GTX-11m at 2.5 and 5 μM was able to partially prevent SMAD2 and SMAD3 activation by TGFβ in a dose-dependent manner (pSMAD2 p = 0.009, p = 0.003; pSMAD3 p = 0.046, p = 0.023). Tendencies to downregulate the protein expression levels of both SMAD2 and 3 were also observed (ns, except SMAD3 GTX-11m 5 μM p < 0.001) (Figure 4B).

We next assessed the effects of GTX-11m over the strong transcriptional reprogramming that follows TGFβ activation in fibroblasts. TGFβ induced the expression of downstream fibrotic markers such as *ACTA2* and *COL1A1* (p = 0.100, p = 0.011)*.* GTX-11m at 5 μM was able to prevent these inductions (p = 0.010, p = 0.017). GTX-11m also showed a tendency to reduce *TNC, MMP2* and *FN* (p = 0.495, p = 0.261, p = 0.036), whereas it tended to increase *MMP1* (p = 0.247) (Figure 4C; Supplementary Figure S4B). The capacity of GTX-11m to prevent the production of ECM components by human fibroblasts was further confirmed at the protein level. Indeed, FN and αSMA induction by TGFβ (p = 0.007, p = 0.017) was reduced by GTX-11m in a dose-dependent manner (GTX-11m 5 μM p = 0.024, p = 0.006) (Figure 4E). Protein secretion of pro-COL1 and FN was also significantly reduced (GTX-11m 2.5 μM p = 0.023, p = 0.013) (Supplementary Figure S4C). GTX-11m tended to induce the expression of N-cadherin (*CDH2*), while it was able to reduce the expression of cadherin 11 (*CDH11*), both at gene (GTX-11m 5 μM p = 0.124, p = 0.037) and protein levels (GTX-11m 5 μM p = 0.048, p < 0.001), indicating its capacity to prevent FMT (Figures 4D,F). Consistently, GTX-11m seemed to induce the expression of forkhead box protein F1 (*FOXF1*) (GTX-11m 5μM p = 0.732) (Figure 4D). In addition, we measured the effects of GTX-11m on hNLFs migration using wound healing assays. While TGFβ alone had no effect over hNLFs migration (p = 0.926), GTX-11m showed a tendency to reduce hNLFs migration (p = 0.324) (Supplementary Figure S4D).

### 3.5 GTX-11m attenuates TGFβ activation in human lung fibroblasts derived from ILD patients

**FIGURE 5:**
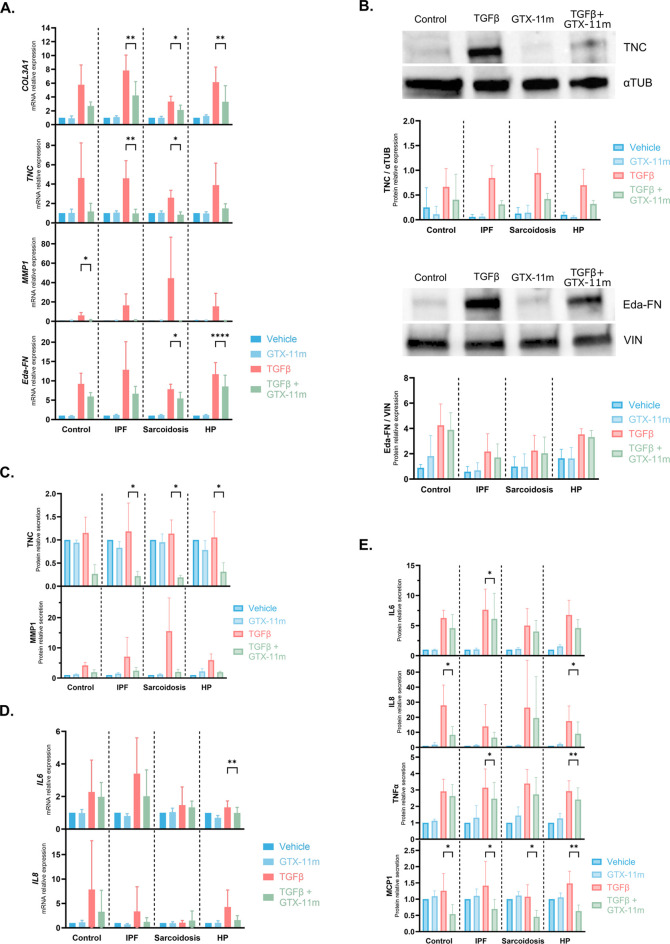
Pro-fibrotic and pro-inflammatory responses to TGFβ treatment in ILD patient-derived fibroblasts are downregulated by GTX-11m. **(A)** Expression of *COL3A1*, *TNC*, *MMP1* and *Eda-FN* was assessed by qPCR in fibroblasts derived from donor lungs after 72 h of vehicle, TGFβ, GTX-11m or combination treatments. **(B)** Protein levels of TNC and Eda-FN in fibroblasts were measured by Western blot after treatments. Upper panels, representative images; lower panels, quantitation of all donors. **(C)** A multiplex immunoassay was used to determine secreted levels of TNC and MMP1 after treatments. **(D)** Expression of *IL6* and *IL8* was assessed by qPCR in fibroblasts derived from donors after treatment with vehicle, GTX-11m, TGFβ alone and TGFβ plus GTX-11m. **(E)** A multiplex immunoassay assay was used to determine secreted levels of IL6, IL8 and TNFα; and ELISA was used to determine secreted levels of MCP1 after treatments. IPF, Idiopathic pulmonary fibrosis; HP, Hypersensitivity pneumonitis. Control donors n = 4, ILD patients n = 5–7. Bars show mean ± SD. Either two-way ANOVA or mixed-effects analysis with Dunnett’s correction for multiple comparisons was performed. Data was baseline normalized in all datasets except for Western blot data. *p < 0.05, **p < 0.01.

To better assess the potential of GTX-11 as a therapeutic drug for lung fibrosis and increase the translatability and robustness of the present study, we used human lung fibroblasts derived from ILD patients and control donors. TGFβ incubation for 72h elicited a strong induction in the expression of *COL3A1*, *TNC*, *MMP1* and FN extra domain A (*eda-FN*). Treatment with GTX-11m effectively prevented these inductions in all disease subtypes (IPF p = 0.001, p = 0.002, p = 0.052, p = 0.065; sarcoidosis p = 0.012, p = 0.012, p = 0.17, p = 0.022; HP p = 0.001, p = 0.120, p = 0.135, p < 0.001), as well as in controls (p = 0.191, p = 0.189, p = 0.048, p = 0.103) (Figure 5A). *COL1A1* and *VEGF* were similarly induced by TGFβ, but GTX-11m treatment did not prevent their inductions (IPF p = 0.562, p = 0.150; sarcoidosis p = 0.903, p = 0.206; HP p = 0.120, p = 0.796; control p = 0.992, p = 0.747). On the other hand, *VIM*, which was not induced by TGFβ, appeared to be downregulated by GTX-11m (IPF p = 0.009, sarcoidosis p = 0.323, HP p = 0.148, control p = 0.178) (Supplementary Figure S5A).

Intracellular protein levels of TNC and FN were then assessed in these cells. While GTX-11m tended to reduce TGFβ-induced intracellular TNC levels in all ILDs (IPF p = 0.267, sarcoidosis p = 0.642, HP p = 0.267, control p = 0.735), no changes were observed in intracellular FN (IPF p = 0.148, sarcoidosis p = 0.456, HP p = 0.269, control p = 0.423) (Figure 5B).

Levels of secreted TNC, MMP1, VEGFα, FN, TIMP1, pro-COL1 and COL3 were also measured in supernatants from ILD patients’ fibroblasts. We observed a significant decrease in secreted TNC when cells were incubated with both TGFβ and GTX-11m (IPF p = 0.012, sarcoidosis p = 0.012, HP p = 0.046, control p = 0.064). Treatment with TGFβ upregulated MMP1 secretion, and GTX-11m showed a trend to reduce it (IPF p = 0.160, sarcoidosis p = 0.309, HP p = 0.059, control p = 0.144) (Figure 5C). A similar pattern was observed when analyzing VEGFα and FN, although reductions were not significant. On the contrary, GTX-11m increased TIMP1 (IPF p = 0.130, sarcoidosis p = 0.304, HP p = 0.259, control p = 0.045) and pro-COL1 (IPF p < 0.001, sarcoidosis p = 0.122, HP p = 0.035, control p = 0.168) secretion when incubated with TGFβ. The same non-significant trend was observed for COL3 (Supplementary Figure S5B) (IPF p = 0.267, sarcoidosis p = 0.421, HP p = 0.356, control p = 0.721).

Emerging evidence suggests that lung-derived fibroblasts play roles in ILD that go beyond structural maintenance and remodeling; certain fibroblast subsets have been shown to critically influence the surrounding immune environment, initiating or amplifying immune signaling pathways (Ghonim et al., 2023). Reflecting this immunomodulatory capacity, ILD patient-derived fibroblasts stimulated with TGFβ for 72h expressed *IL6* and *IL8*. Importantly, GTX-11m showed a trend toward mitigating these inductions (*IL6*: IPF p = 0.340, sarcoidosis p = 0.956, HP p = 0.009, control p = 0.941; *IL8*: IPF p = 0.567, sarcoidosis p = 0.897, HP p = 0.204, control p = 0.405) (Figure 5D). A broader panel of cytokines and chemokines was subsequently analyzed in culture supernatants. Here, IL-6, IL-8, TNFα and MCP1 were all induced by TGFβ in both ILD and control fibroblasts. GTX-11m effectively suppressed these inductions, reaching statistical significance in the indicated conditions (IL6 IPF p = 0.041; IL8 HP p = 0.016, control p = 0.038; TNFα IPF p = 0.017, HP p = 0.006; MCP1 IPF p = 0.027, sarcoidosis p = 0.023, HP p = 0.004, control p = 0.023) (Figure 5E).

### 3.6 Comparative effects of GTX-11m, nintedanib and pirfenidone on TGFβ-induced pro-fibrotic gene expression in hNLFs

Nintedanib and pirfenidone are two current approved drugs for IPF treatment. Both have been reported to exert some of their antifibrotic effects directly on the fibroblast population. We thus aimed to compare the above observed effects of GTX-11m on hNLFs to those of these two drugs. In this set of experiments, R-268712, an inhibitor of the TGFβ type I receptor kinase (ALK5), served as an inhibition control of the TGFβ pathway.

**FIGURE 6:**
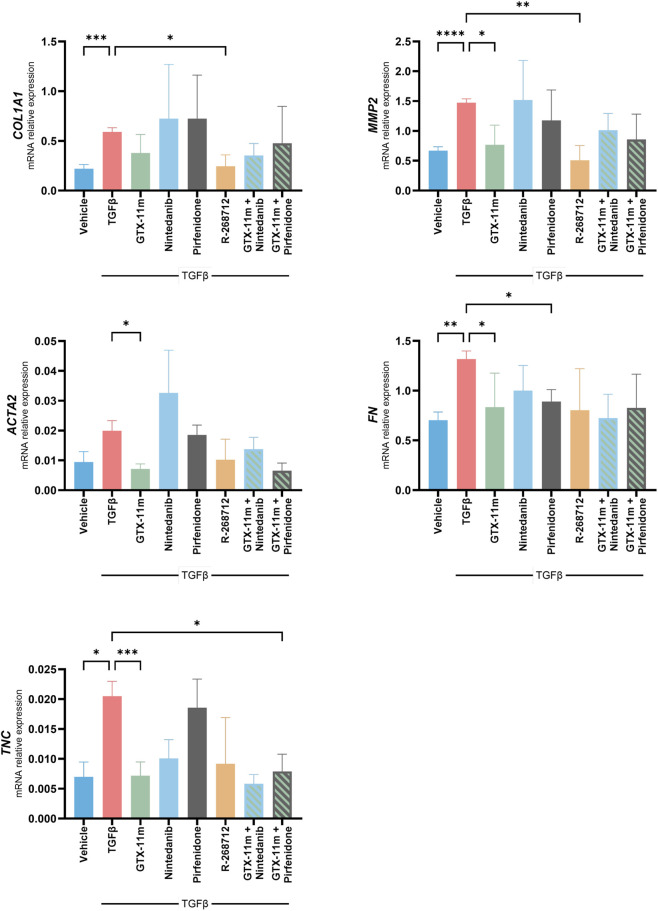
Comparative effects of GTX-11m and approved antifibrotic therapies on fibrotic marker induction in hNLFs. Gene expression levels of relevant fibrotic markers *COL1A1, MMP2, ACTA2, FN and TNC*, after incubations with TGFβ1 and GTX-11m (5 μM), nintedanib (1 μM), pirfenidone (1 mM), R-268712 (1 μM) or the indicated combinations for 48h (n = 5). Bars show mean ± SD. Mixed-effects analysis with Dunnett’s correction for multiple comparisons was performed in all graphs. *p < 0.05, **p < 0.01, ***p < 0.001, ****p < 0.0001.

In hNLFs, neither nintedanib nor pirfenidone prevented the TGFβ-induced expression of *COL1A1* (p = 0.984, p = 0.944), while GTX-11m showed an inhibitory trend (p = 0.101). This trend was maintained when GTX-11m was used in combination with either of the two drugs (p = 0.084, p = 0.967). GTX-11m demonstrated good inhibition capacity of *MMP2* and *ACTA2* (p = 0.016, p = 0.013), which was not matched by the approved drugs, and this ability persisted in the combination treatments (nintedanib p = 0.260, p = 0.302; pirfenidone p = 0.127, p = 0.080). While all drugs partially prevented *FN* induction, pirfenidone and GTX-11m (p = 0.027, p = 0.044) were more effective than nintedanib (p = 0.200). GTX-11m and nintedanib showed similar capacities to prevent *TNC* induction by TGFβ (p < 0.001, p = 0.189), whereas pirfenidone was ineffective (p = 0.991) (Figure 6). No synergistic effect between GTX-11m and either nintedanib or pirfenidone was observed for any gene across the tested treatment combinations. Direct ALK5 inhibition proved to downregulate gene expression in all these cases, although not always statistically significant. Aside from that, *MMP1* was not induced by TGFβ in hNFLs (p = 0.954) (Supplementary Figure S6).

## 4 Discussion

GTX-11 is an orally bioavailable compound in clinical development for lung fibrotic diseases. Most ILDs are associated with high mortality, and current therapeutic options are limited. The well-established single-dose bleomycin-induced lung fibrosis model is the standard animal model for these diseases, as recommended by the American Thoracic Society (B. Moore et al., 2013). We used this model to prove the potential of GTX-11 as a therapeutic drug for lung fibrosis. Remarkably, GTX-11 improved animal survival and significantly reduced lung fibrosis as measured by Ashcroft score and collagen deposition. Analysis of the expression of relevant fibrotic markers supported the antifibrotic effects of GTX-11.

GTX-11 also lowered the inflammatory cell count in BALF by reducing the populations of macrophages, neutrophils and lymphocytes. Significant reductions in lung tissue mRNA levels of the proinflammatory cytokines *Tnfa* and *Il6,* and of the chemoattractant *Mcp1* further confirm the anti-inflammatory properties of GTX-11. Importantly, GTX-11 treatment was administered starting on day 10 after the bleomycin instillation, past the inflammatory phase of the disease and once fibrosis had been established (Kolb et al., 2020). It is thus unlikely that the broad antifibrotic effects of GTX-11 are merely due to its anti-inflammatory capacities.

Additionally, GTX-11 greatly reverted lung vascular dysfunction, reduced pulmonary vascular remodeling and normalized the altered bleomycin-induced expression values of endothelial markers. Furthermore, GTX-11 restored abnormal right ventricular systolic pressure and volume to control values, indicating an overall improvement in cardiac function. These effects could be attributed to a direct action of GTX-11 over the vascular tissue or to a paracrine effect reflecting the overall improvement of these animals.

In the fibrotic lung, fibroblasts are the major cellular component of the stroma and drive disease progression through their transition to myofibroblasts, which are the primary effectors of fibrotic tissue remodeling (Ghonim et al., 2023). In hNLFs, GTX-11m was able to modulate the TGFβ pathway by significantly reducing SMAD2 and SMAD3 phosphorylation. This was accompanied by a reduction in the induction of mRNA of critical fibrotic markers *COL1A1*, *ACTA2* and *TNC*, and corroborated by diminished induction of protein levels of FN and αSMA. Furthermore, GTX-11m treatment prevented the switch from cadherin 2 to cadherin 11, a change that flags the conversion to myofibroblast.

ILD patient-derived fibroblasts represent another valuable tool for validating the potential effects of novel therapeutics. Here, high patient variability in basal expression levels made inducibility a more robust readout than absolute expression. Fibroblasts from the three evaluated ILDs–sarcoidosis, hypersensitivity pneumonitis and idiopathic pulmonary fibrosis–retained their capacity to respond both to TGFβ and GTX-11m. In these fibroblasts, GTX-11m was able to prevent the production of relevant fibrotic molecules, such as TNC and FN, as well as the induction of pro-inflammatory cytokines IL6 and IL8. These positive results in a relevant cell model pave the road for further development of GTX-11 as an antifibrotic.

In our model of bleomycin-induced lung fibrosis, nintedanib produced similar effects on fibrotic gene expression and on Ashcroft score to the 2 mg/kg dose of GTX-11. It also exerted mild, non-significant effects on the inflammatory component of the disease, consistent with previous reports (Ackermann et al., 2017). On the other hand, nintedanib effectively reduced some of the endothelial alterations induced by bleomycin, probably due to its intrinsic PDGF and VEGF inhibition activities. Yet, GTX-11m demonstrated greater efficacy across most of the assessed endothelial readouts. Hence, we hypothesized that nintedanib may benefit from a co-treatment regimen with GTX-11. We subsequently tested GTX-11m effectiveness in preventing hNLFs activation by TGFβ in comparison and in combination with the approved drugs nintedanib and pirfenidone. In this setting, GTX-11m effects on fibrotic gene expression in lung fibroblasts were more consistent than those of pirfenidone or nintedanib, but were comparable in strength to those observed with direct ALK5 inhibition using R-268712. These findings are consistent with our previous studies on pirfenidone (Molina-Molina et al., 2018) and support the notion that pirfenidone alone may not strongly modulate certain key fibrotic genes, particularly when tested in isolated fibroblast models without the full tissue context. However, direct comparison between compounds should be interpreted with caution due to key pharmacological differences, including different mechanisms of action and different pharmacodynamic profiles (intracellular accumulation, metabolic stability, onset, duration, and timing of peak activity), which may influence their apparent activity in short-term *in vitro* settings.

Although TGFβ plays pivotal roles in keeping tissue homeostasis, immune balance and wound healing; dysregulated TGFβ activation following injury or other insults is thought to lead to aberrant wound healing and fibrotic disease in the lungs and other organs (Deng et al., 2024). Here we show that, in lung fibroblasts, GTX-11m decreases SMAD2/3 phosphorylation and the transcription of downstream profibrotic genes, thereby limiting fibroblast-to-myofibroblast transition and excessive ECM deposition. However, in the lungs, TGFβ acts far beyond fibroblasts. In the immune system, it helps maintain homeostasis and resolve inflammation, though overactivation can drive harmful immune responses (Batlle and Massagué, 2019). Precisely we have shown that GTX-11m reduces TGFβ-induced secretion of pro-inflammatory cytokines, an anti-inflammatory effect that likely reduces immune-fibroblast cross-activation. Finally, in endothelial cells, which also express TGFβ receptors, TGFβ is critical for angiogenic development and wound healing processes. While complete inhibition of the pathway is toxic, targeting pathological TGFβ overactivation can restore vascular balance by modulating adhesion molecules, limiting endothelial–mesenchymal transition (EndMT), and reestablishing vascular homeostasis (Deng et al., 2024). In our bleomycin model, GTX-11 promotes the restoration of endothelial integrity and vascular homeostasis, an effect that may be partially mediated by its modulatory activity on the TGFβ signaling pathway. Additionally, GTX-11m rebalanced the production of cadherins 2 and 11, a process regulated by the transcription factor FOXF1, which has been widely reported to exert anti-fibrotic effects. Mechanistically, in lung fibroblasts FOXF1 inhibits the pro-fibrotic TGFβ pathway, potentially through a direct downregulation of SMAD2/3 activation. In parallel, in epithelial cells FOXF1 negatively regulates Wnt/β-catenin signaling. In both fibroblasts and adult lung endothelial cells, FOXF1 acts through direct transcriptional regulation of specific cadherins CDH2 and CDH11, or CDH5. Through these mechanisms, FOXF1 maintains endothelial barrier function, prevents myofibroblast differentiation and fibrogenesis, and modulates profibrotic and inflammatory responses. Consistent with these functions, FOXF1 expression is reduced in both IPF fibroblasts and endothelial cells, thereby compromising its antifibrotic function (Melboucy-Belkhir et al., 2014; Cai et al., 2016; Black et al., 2018; Bian et al., 2023; Chen et al., 2023; Hu et al., 2024). Here, we report that GTX-11m prevented TGFβ-induced SMAD activation and tended to elevate the mRNA levels of FOXF1, although further experiments are needed to fully characterize both pathways. These direct effects on fibroblasts might largely account for the antifibrotic effects observed with GTX-11 in the bleomycin rat model, providing preliminary evidence of translatability from the animal model to the human context.

Here, we show for the first time that direct administration of GTX-11 is enough to mimic the therapeutic effects of FX in the fibrotic lung. To notice, higher doses are required for FX to parallel GTX-11 effects, presumably due to lower bioavailability and the need of conversion to its pharmacologically active metabolites (Asai et al., 2004; Mok et al., 2018; Li et al., 2021; Stiefvatter et al., 2021; Takatani et al., 2022). Additionally, preclinical toxicological studies with GTX-11 show that it has a broad therapeutic window, an excellent safety profile and that it causes none of the commonly described side effects associated with TGFβ inhibition, such as cardiac or skin toxicity (Ciardiello et al., 2020; Peng et al., 2022). Altogether, it has led to a Phase I study in humans (EU CT 2023–504426-19–00). We thus propose that the administration of GTX-11 is a suitable therapeutic alternative to the administration of the parental FX with an enhanced bioavailability profile.

Overall, the antifibrotic efficacy of GTX-11 has been validated across three different models, demonstrating a high level of consistency, with discrepancies observed in only a small number of readouts. Despite the robustness of the data presented, this study presents several limitations. First, although we show the capacity of GTX-11m to prevent TGFβ-induced SMAD activation, the precise molecular mechanism of this interference remains to be fully characterized, and the direct binding partners of the compound have yet to be unequivocally identified. In this line, even if we demonstrated GTX-11m rebalancing effect on Cadherins 2 and 11, the ability to modulate FOXF1 still requires further validation and mechanistic investigation. Finally, although we demonstrated consistent antifibrotic activity *in vitro* and *in vivo*, these models do not fully recapitulate the chronic and heterogeneous nature of human IPF. To address these limitations, current studies are ongoing to deepen our understanding of the effects of GTX-11 on human lung immune and endothelial cells, as well as to validate GTX-11m therapeutic effects in human healthy and ILD-derived precision-cut lung slices.

In conclusion, we found that GTX-11 is an effective antifibrotic *in vivo,* with beneficial effects over the three main axes of disease: fibrosis, inflammation and endothelial dysfunction. Importantly, when compared to nintedanib, GTX-11 exerts similar or better effects across all measured parameters. We were able to validate the antifibrotic effects of GTX-11 *in vitro* in human fibroblasts, and further confirmed these effects in ILD-derived fibroblasts. Together, these results highlight the therapeutic potential of GTX-11 as a novel approach to fibrotic lung disease. Ongoing clinical trials will be critical to confirming its safety in humans, with future studies planned to assess its efficacy as a treatment for pulmonary fibrosis.

## Data Availability

The raw data supporting the conclusions of this article will be made available by the authors, without undue reservation.

## Glossary

αSMA: α Smooth Muscle Actin (ACTA2)
αTUB: α Tubulin
ACX: Amarouciaxanthin A
ALK5: Activin Receptor-Like Kinase 5 (TGFβ type I receptor)
ANOVA: Analysis of Variance
BALF: Bronchoalveolar Lavage Fluid
BLM: Bleomycin
BrdU: Bromodeoxyuridine
CDH2: Cadherin 2 (N-cadherin)
CDH5: Cadherin 5 (VE-cadherin)
CDH11: Cadherin 11
COL1: Collagen 1
COL3: Collagen 3
Ct: Threshold Cycle
CTGF: Connective Tissue Growth Factor
EA: External Area
ECM: Extracellular Matrix
ED: External Diameter
EDN1: Endothelin 1
EndMT: Endothelial–Mesenchymal Transition
EP: External Perimeter
FMT: Fibroblast-to-Myofibroblast Transition
FN: Fibronectin
FOV: Field of view
FOXF1: Forkhead Box F1
FX: Fucoxanthin
Fxol: Fucoxanthinol
GAPDH: Glyceraldehyde-3-Phosphate Dehydrogenase
hNLFs: Human Normal Lung Fibroblasts
HP: Hypersensitivity Pneumonitis
IA: Internal Area
ICAM1: Intercellular Adhesion Molecule 1
ID: Internal Diameter
IL6: Interleukin 6
IL8: Interleukin 8
ILDs: Interstitial Lung Diseases
IPF: Idiopathic Pulmonary Fibrosis
IVIS: *In Vivo* Imaging System
LHV/LV: Left Heart Ventricle/Left Ventricle
MASH: Metabolic Dysfunction-Associated Steatohepatitis
MCP1: Monocyte Chemoattractant Protein 1
MMP: Matrix Metalloproteinase
MRI: Magnetic Resonance Imaging
NOX4: NADPH Oxidase 4
PBS: Phosphate Buffered Saline
PDGFb: Platelet-Derived Growth Factor Subunit B
PECAM1: Platelet Endothelial Cell Adhesion Molecule 1 (CD31)
PVDF: Polyvinylidene difluoride
qPCR: Quantitative real-time PCR
RHV/RV: Right Heart Ventricle/Right Ventricle
RVSP: Right Ventricular Systolic Pressure
SD: Standard Deviation
Sele: E-selectin
TGFβ: Transforming Growth Factor β
TIMP1: Tissue Inhibitor of Metalloproteinase 1
TNC: Tenascin C
TNFα: Tumor Necrosis Factor α
VEGF: Vascular Endothelial Growth Factor
VIM: Vimentin
VIN: Vinculin
WA: Wall Area
WT: Wall Thickness
