# Supplementary material for: GTX-11 attenuates lung fibrosis, inflammation and vascular remodeling in preclinical models of lung fibrotic disease
Source: Front Pharmacol. 2026 Jan 27;16:1671132. doi: 10.3389/fphar.2025.1671132 (PMC12887705; doi:10.3389/fphar.2025.1671132)
Supplement: Supplementary file 1 [file DataSheet1.docx]

Supplementary Material

# Graphical abstract


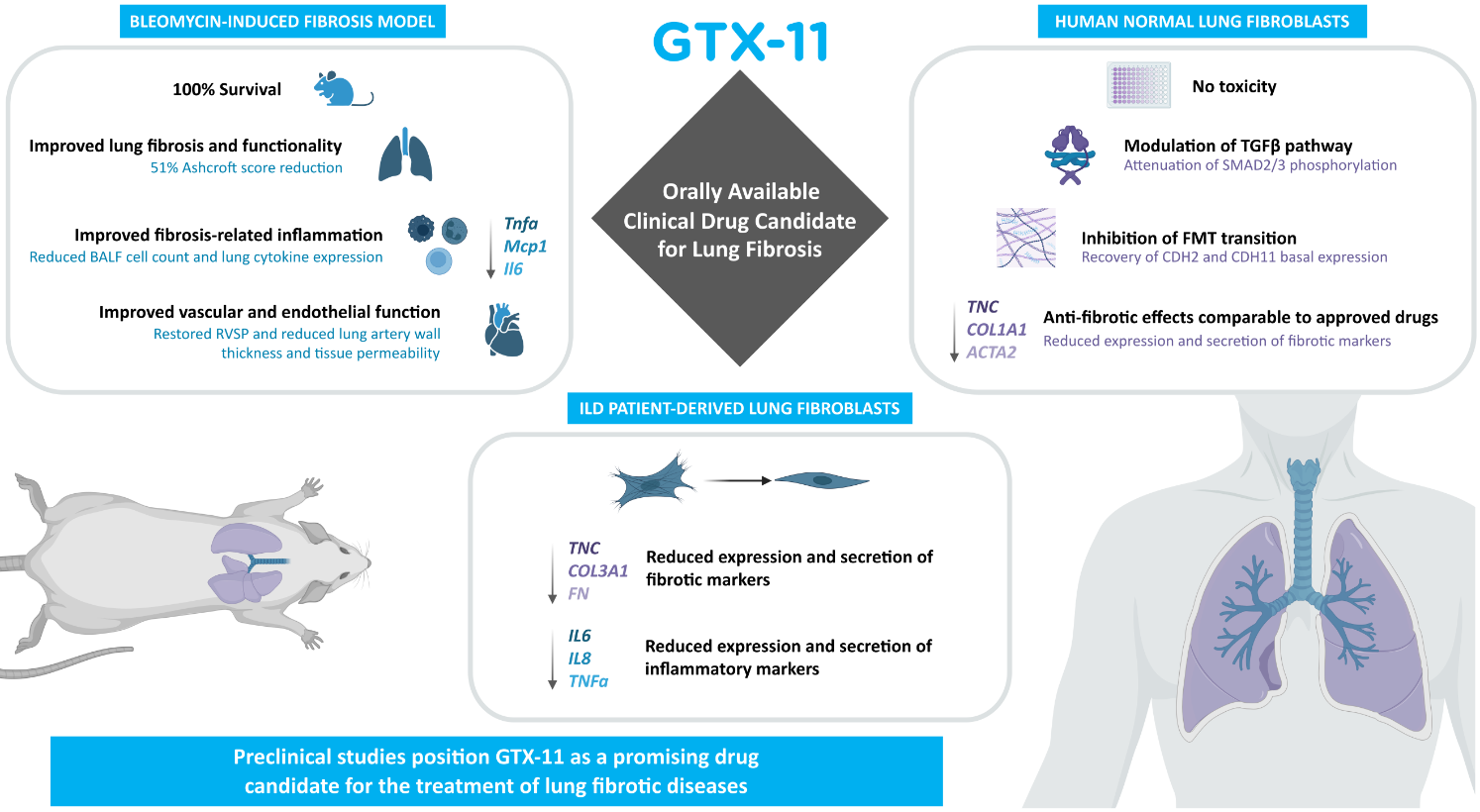


# Supplementary Figures


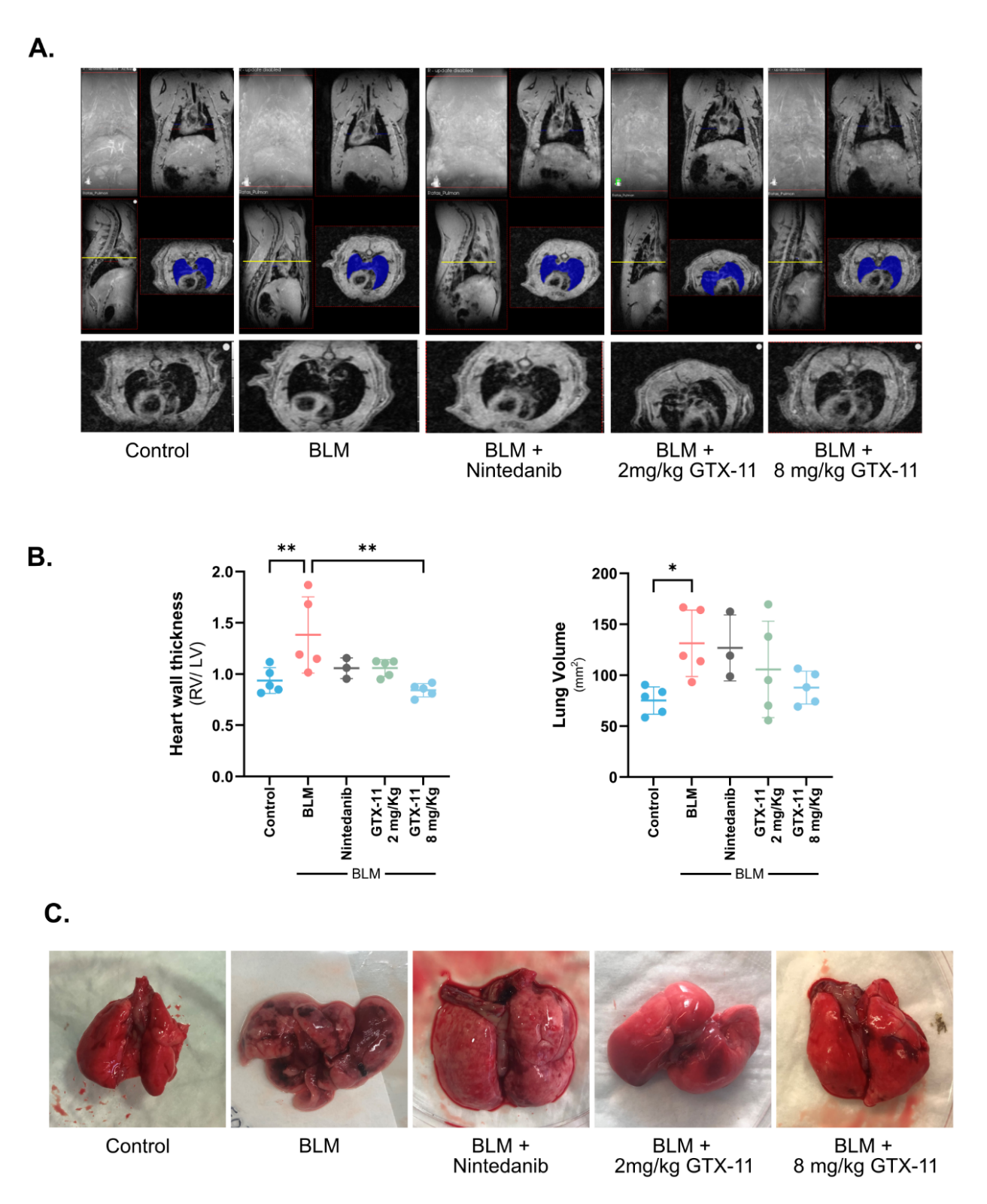


## Supplementary Figure 1. Macroscopic effects of GTX-11 administration in a rat model of bleomycin-induced lung fibrosis.

**(A)** Representative images of lung section analysis by RMI from each treatment group. **(B)** Lung volume and heart wall thickness determination by RMI (n=5). **(C)** Representative images of lung appearance at the time of sacrifice from each treatment group.

Bars show mean ± SD. One way ANOVA with Dunnett’s correction for multiple comparisons was performed. *p<0.05, ** p<0.01.


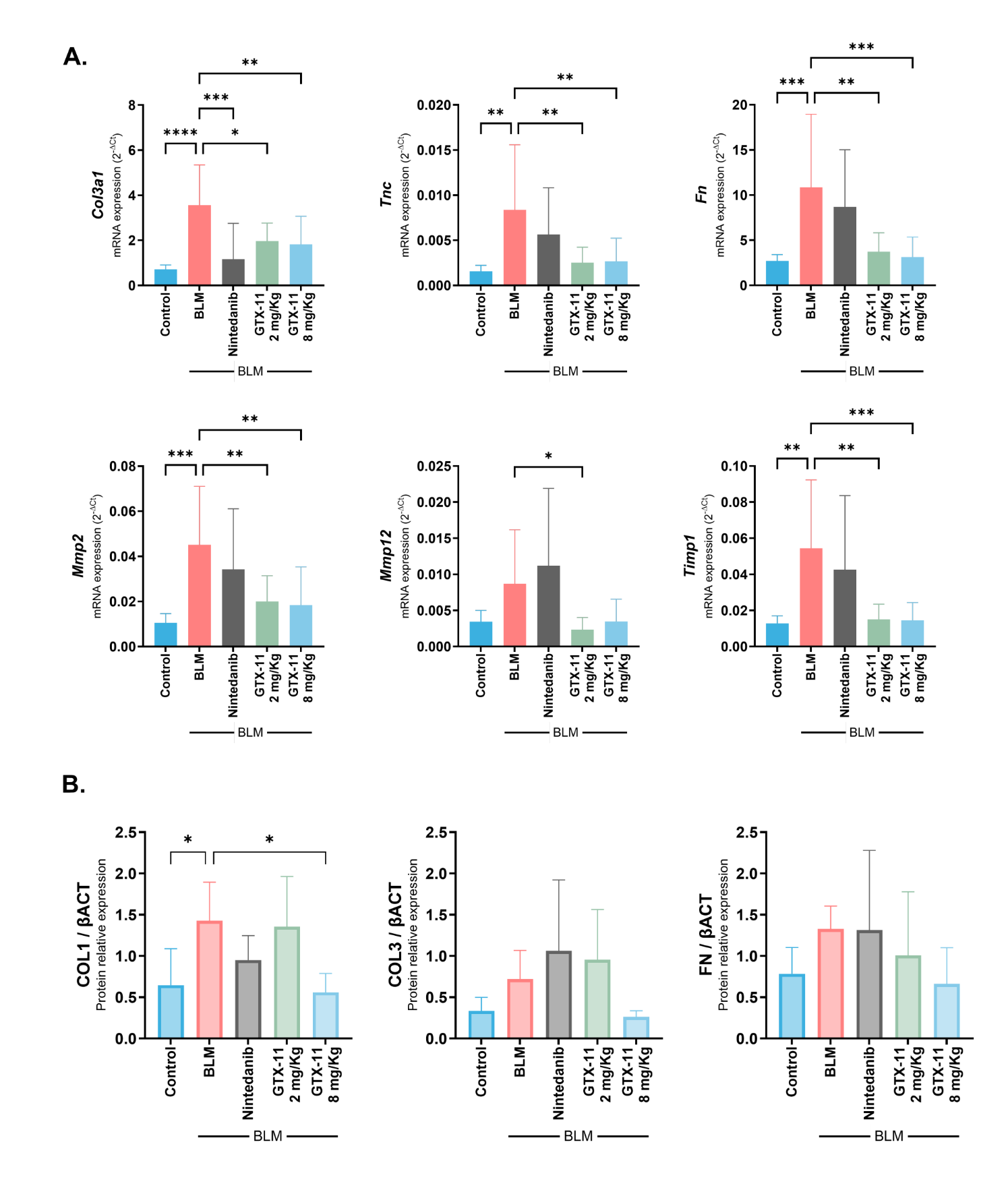


## Supplementary Figure 2. Effects of GTX-11 on additional fibrotic markers in a rat model of bleomycin-induced lung fibrosis.

**(A)** Gene expression of fibrotic markers *Col3a1, Tnc, Fn, Mmp2, Mmp12* and *Timp1* in lung tissue lysates was measured by qPCR. **(B)** Protein levels of COL1, COL3 and FN in lung tissue lysates, as detected by Western blot. βACT, β actin.

Bars show mean ± SD. One way ANOVA with Dunnett’s multiple comparison test was performed in all graphs. *p<0.05, ** p<0.01, *** p<0.001, **** p<0.0001


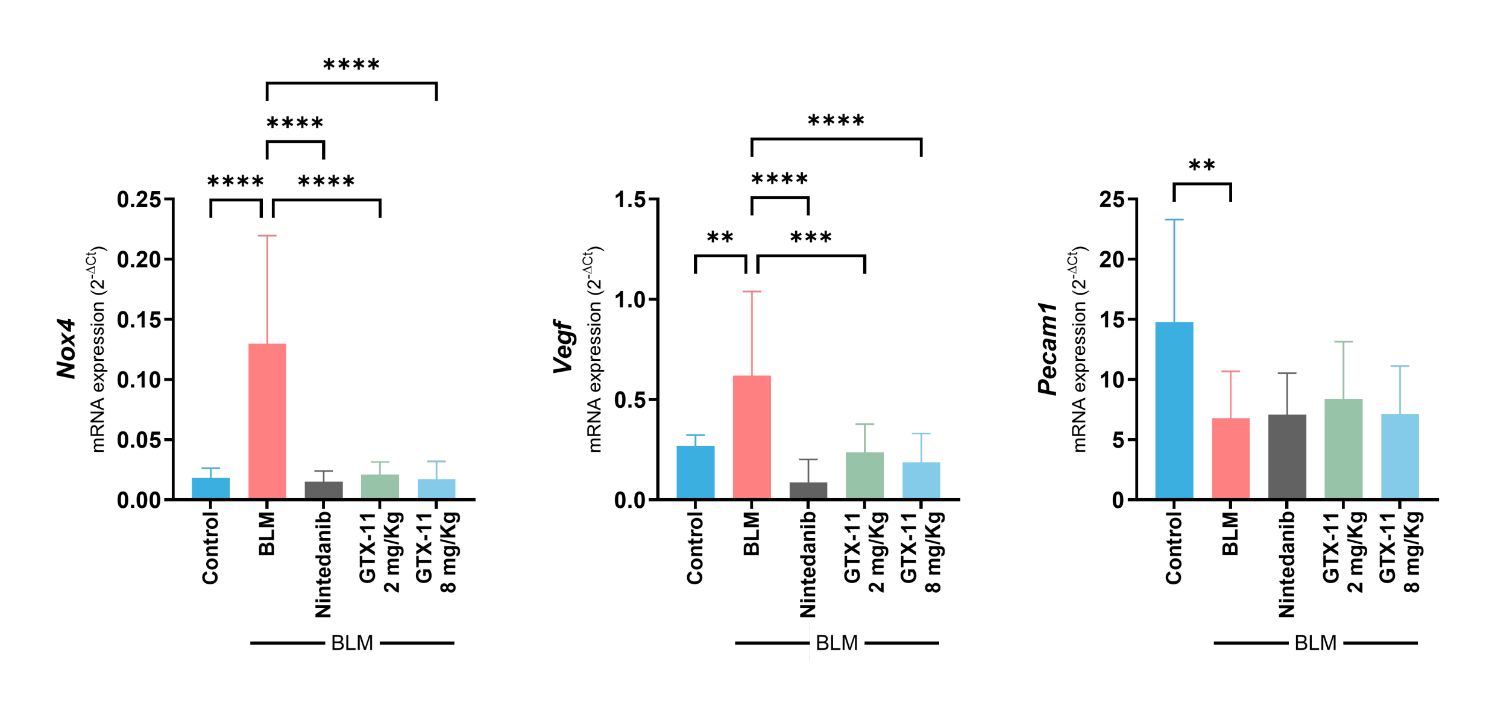


## Supplementary Figure 3. Effects of GTX-11 in additional endothelial markers in a rat model of bleomycin-induced lung fibrosis.

Gene expression of endothelial markers Nox4, Vegf and Pecam1 in lung tissue lysates, as measured by qPCR.

Bars show mean values ± SD. One way ANOVA with Dunnett’s multiple comparison correction was performed in all graphs. ** p<0.01, *** p<0.001, **** p<0.0001


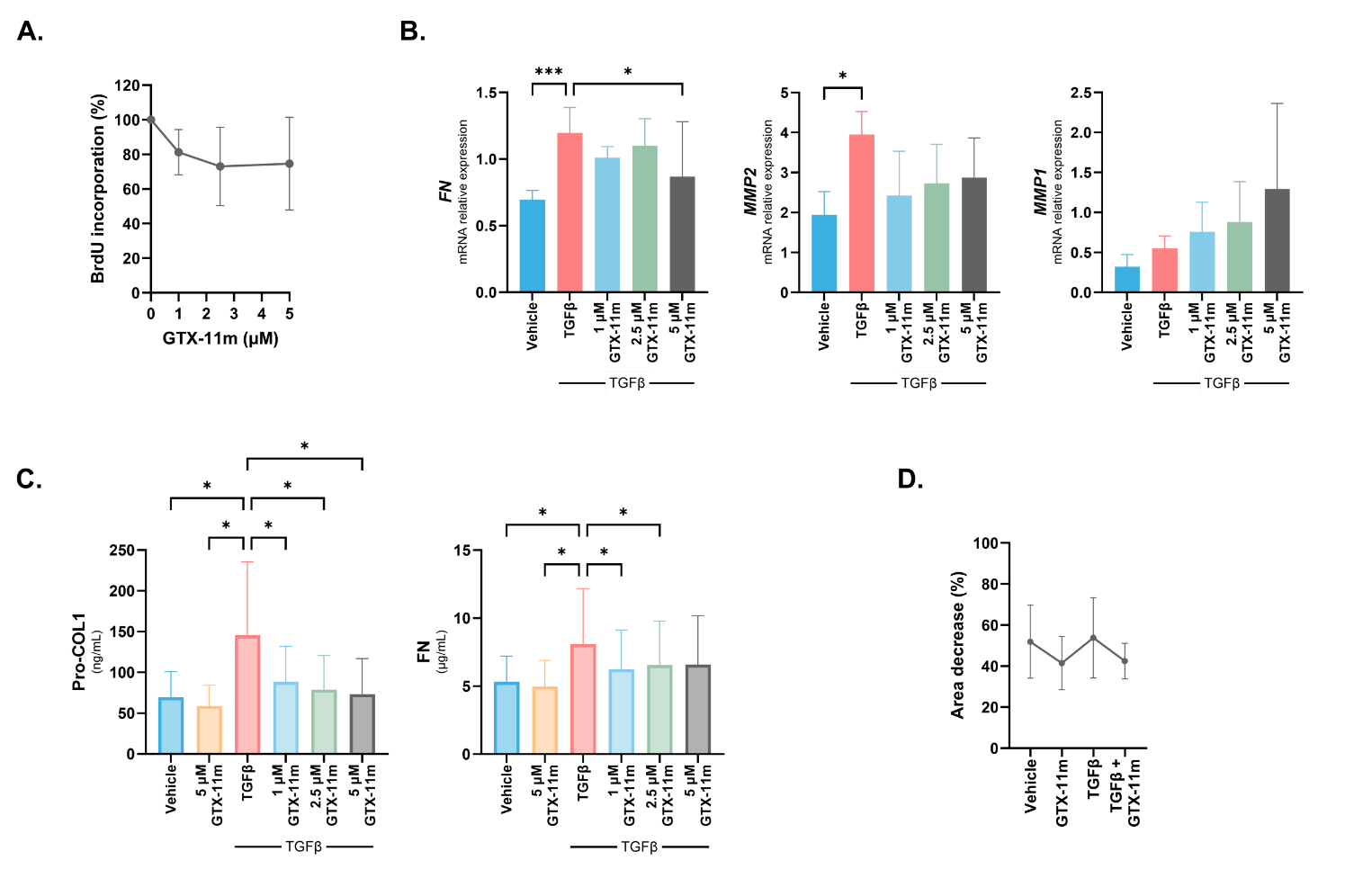


## Supplementary Figure 4. Additional findings on the effect of GTX-11m on hNLFs.

**(A)** BrdU incorporation assays were performed at different GTX-11m concentrations for 48 hours to assess cell proliferation. Data is represented as percent change relative to vehicle (n=7). **(B)** Gene expression of fibrotic markers *FN, MMP2* and *MMP1* in hNLFs was measured by qPCR after TGFβ1 and GTX-11m treatment for 48 hours (n=5). **(C)** hNLFs secreted levels of pro-COL1 and FN were measured after 48 hours of treatments with ELISA (n=4). **(D)** The effect of TGFβ1 and GTX-11m in hNLFs migration was measured in wound healing assays (n=4).

Bars and lines show mean ± SD. One way ANOVA with Dunnett’s multiple comparison test was performed in all graphs. *p<0.05, *** p<0.001


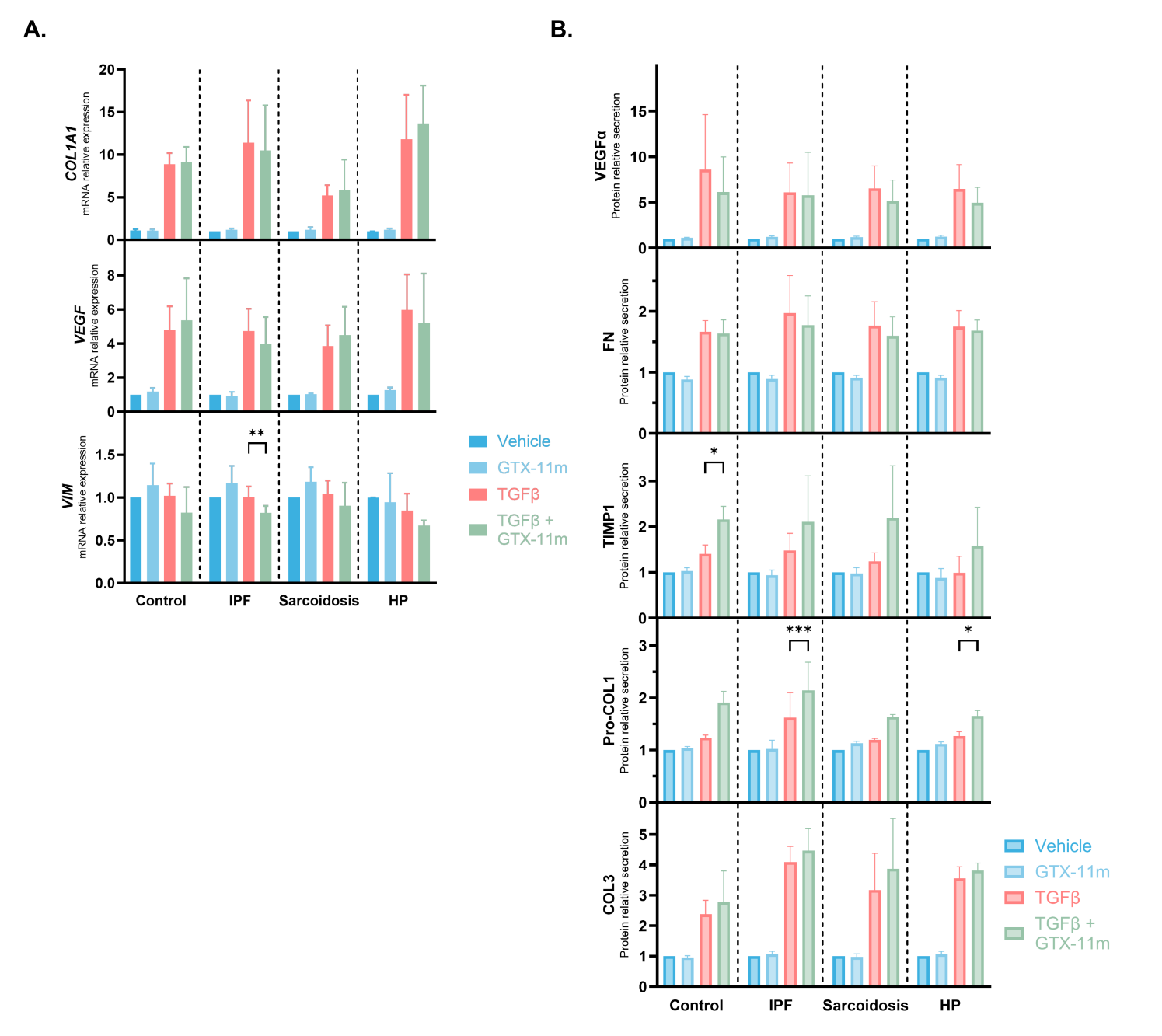


## Supplementary Figure 5. Effects of GTX-11m in additional fibrotic markers in human lung fibroblasts derived from ILD patients.

**(A)** Gene expression of fibrotic markers *COL1A1, VEGF* and *VIM* in hNLFs was measured by qPCR in fibroblasts derived from donors after treatment with vehicle, GTX-11m, TGFβ alone and TGFβ plus GTX-11m. **(B)** A multiplex immunoassay assay was used to determine secreted levels of VEGF and TIMP1; and ELISA was used to determine secreted levels of FN, pro-COL1 and COL3 after treatments.

IPF, Idiopathic pulmonary fibrosis; HP, Hypersensitivity pneumonitis.

In general, control donors n=4, ILD patients n=5-7. In pro-COL1 and COL3 measurements, n=2 for control donors and all ILDs except IPF, where n=7. Bars show mean ± SD. Two-way ANOVA or mixed-effect analysis with Dunnett’s multiple comparison correction were performed. **p*<0.05, ** p<0.01, *** p<0.001


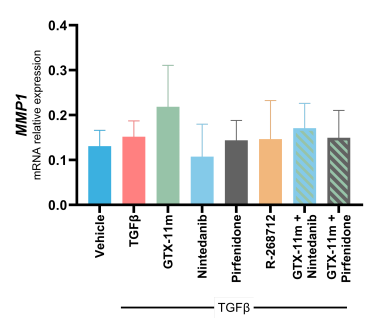


## Supplementary Figure 6. Comparative effects of GTX-11m and approved antifibrotic therapies on MMP1 expression in hNLFs.

Gene expression levels of MMP1 after incubations with TGFβ1 and GTX-11m (5 μM), nintedanib (1 μM), pirfenidone (1 mM), R-268712 (1 μM) or the indicated combinations for 48h (n=5).

Bars show mean ± SD. A mixed-effects analysis with Dunnett’s multiple comparison correction was performed.

# Supplementary Tables

## Supplementary Table 1. List of primary antibodies used in Western blotting.

|  | **Target protein** | **Source** | **Identifier** |
| --- | --- | --- | --- |
| **Rat** | **βActin** | Sigma-Aldrich | A5441 |
|  | **COL1** | Cell Signaling | 84336S |
|  | **COL3** | Proteintech | 22734-1-AP |
|  | **Fibronectin** | Proteintech | 15613-1-AP |
| **Human** | **αSMA** | ScyTek | Clone 1A4  Cat# A00002-C |
|  | **Actin** | Sigma-Aldrich | Polyclonal  Cat# A2066 |
|  | **CDH2** | Santa Cruz Biotechnology | Clone 13A9  Cat# sc-59987 |
|  | **CDH11** | Santa Cruz Biotechnology | Clone F-3  Cat# sc-365867 |
|  | **Eda-FN** | Abcam | ab125233 |
|  | **Fibronectin** | Santa Cruz Biotechnology | Clone EP5  Cat# sc-8422 |
|  | **Phospho-SMAD2 (S465/S467)** | Cell Signaling | Clone E8F3R  Cat# 18338 |
|  | **Phosho-SMAD3**  **(S423/425)** | Cell Signaling | Clone C25A9  Cat# 9520S |
|  | **SMAD2** | Cell Signaling | Clone D43B4  Cat# 5339T |
|  | **SMAD3** | Abcam | Clone EP568Y  Cat# ab40854 |
|  | **TNC** | Abcam | Clone T2H5  Cat# ab3970 |
|  | **Tubulin** | Sigma-Aldrich | Clone DM1A  Cat# T6199 |
|  | **Vinculin** | Abcam | Clone EPR8185  Cat# ab129002 |

## Supplementary Table 2. List of rat qPCR primer pairs.

| **Target gene** | **Source** | **Reference** |
| --- | --- | --- |
| ***Acta2*** | Thermofisher | ***RN01759928_g1*** |
| ***Cdh5*** | Thermofisher | ***RN01536708_m1*** |
| ***Col1a1*** | Thermofisher | ***RN01463848_m1*** |
| ***Col3a1*** | Thermofisher | ***RN01437681_m1*** |
| ***Ctgf*** | Thermofisher | ***RN00573960_m1*** |
| ***Edn1*** | Thermofisher | ***RN00561129_m1*** |
| ***Fn*** | Thermofisher | ***RN00569575_m1*** |
| ***Icam1*** | Thermofisher | ***RN00564227_m1*** |
| ***Il6*** | Thermofisher | ***RN00561420_m1*** |
| ***Mcp1*** | Thermofisher | ***RN00580555_m1*** |
| ***Mmp12*** | Thermofisher | ***RN00588640_m1*** |
| ***Mmp2*** | Thermofisher | ***RN01538170_m1*** |
| ***Nox4*** | Thermofisher | ***RN00585380_m1*** |
| ***Pdgfb*** | Thermofisher | ***RN01502596_m1*** |
| ***Pecam1*** | Thermofisher | RN***01467262_m1*** |
| ***Sele*** | Thermofisher | ***RN00594072_m1*** |
| ***Tgfb1*** | Thermofisher | ***RN00572010_m1*** |
| ***Timp1*** | Thermofisher | ***RN01430873_g1*** |
| ***Tnc*** | Thermofisher | ***RN01454948_m1*** |
| ***Tnfa*** | Thermofisher | ***RN00562055_m1*** |
| ***Vegf*** | Thermofisher | ***RN01511602_m1*** |

## Supplementary Table 3. List of human qPCR primer pairs.

| **Target gene** | **Source** | **Sequence 5’- 3’** |
| --- | --- | --- |
| ***ACTA2*** | IDT Technologies | Fw ACTGCCTTGGTGTGTGACAA  Rv CACCATCACCCCCTGATGTC |
| ***CDH11*** | IDT Technologies | Fw AGAGGTCCAATGTGGGAACG  Rv GGTTGTCCTTCGAGGATACTGT |
| ***CDH2*** | IDT Technologies | Fw CAGTATCCGGTCCGATCTGC  Rv GAGCTGTGGGGTCATTGTCA |
| ***COL1A1*** | IDT Technologies | Fw GCTCCTGCTCCTCTTAGCG  Rv CCGTTCTGTACGCAGGTGAT |
| ***FN*** | IDT Technologies | Fw CTGCAAGCCCATAGCTGAGA  Rv GAAGTGCAAGTGATGCGTCC |
| ***FOXF1*** | IDT Technologies | Fw CACCAGAACAGCCACAACG  Rv CTGCTGGTGGTAGTAGGAGC |
| ***MMP2*** | IDT Technologies | Fw TGGATGATGCCTTTGCTCGT  Rv CAAAGGGGTATCCATCGCCA |
| ***PAI1*** | IDT Technologies | Fw CACCAACATTCTGAGTGCCC  Rv GGTTCTCTAGGGGCTTCCTG |
| ***TNC*** | IDT Technologies | Fw GCATCCGGACCAAAACCATC  Rv TCCAGGAAACTGTGAACCCG |
| ***ACTA2*** | Sigma-Aldrich | Fw CTGTTCCAGCCATCCATCCTTCAT  Rv CCGTGATCTCCTTCTGCATT |
| ***b-ACTIN*** | Sigma-Aldrich | Fw TGCTTGCTGATCCACATCTGCT  Rv GCACTCTTCCAGCCTTCCTTCC |
| ***COL1A1*** | Sigma-Aldrich | Fw CTTCACCTACAGCGTCACTG  Rv GGATGGAGGGAGTTTACAGG |
| ***FN*** | Sigma-Aldrich | Fw TAAAGGACTGGCATTCACTGA  Rv GTGCAAGGCAACCACACTGAC |
| ***GAPDH*** | Sigma-Aldrich | Fw ACCACAGTCCATGCCATCAC  Rv TCCACCACCCTGTTGCTGTA |
| ***HPRT1*** | Sigma-Aldrich | Fw AAGGACCCCACGAAGTGTTG  Rv GGCTTTGTATTTTGCTTTTCCA |
| ***IL6*** | Sigma-Aldrich | Fw CCCCAGGAGAAGATTCCAAA  Rv CCAGTGATGATTTTCACCAGG |
| ***IL8*** | Sigma-Aldrich | Fw CACCGGAAGGAACCATCTCACTGT  Rv TCCTTGGCAAAACTGCACCTTCA |
| ***MMP1*** | Sigma-Aldrich | Fw GGGAGATCATCGGGACAACTC  Rv GGGCCTGGAAGAAAAGCAT |
| ***MMP2*** | Sigma-Aldrich | Fw CGTCTGTCCCAGGATGACATC  Rv ATGTCAGGAGAGGCCCCATA |
| ***TNC*** | Sigma-Aldrich | Fw CGACGTGTTTCCAGACAGA  Rv GTGGCTTGTTGGCTTTGG |
| ***VEGF*** | Sigma-Aldrich | Fw TGCTTCTGAGTTGCCCAGGA  Rv TGGTTTCAATGGTGTGAGGACATAG |
| ***VIM*** | Sigma-Aldrich | Fw GAGAACTTTGCCGTTGAAGC  Rv CTCAATGTCAAGGGCCATCT |
